# Tick communities of cattle in smallholder rural livestock production systems in sub-Saharan Africa

**DOI:** 10.1186/s13071-023-05801-5

**Published:** 2023-06-19

**Authors:** Dieter J. A. Heylen, Bersissa Kumsa, Elikira Kimbita, Mwiine Nobert Frank, Dennis Muhanguzi, Frans Jongejan, Safiou Bienvenu Adehan, Alassane Toure, Fred Aboagye-Antwi, Ndudim Isaac Ogo, Nick Juleff, Josephus Fourie, Alec Evans, Joseph Byaruhanga, Maxime Madder

**Affiliations:** 1 Evolutionary Ecology Group, Department of Biology, University of Antwerp, Wilrijk, Belgium; 2 Interuniversity Institute for Biostatistics and Statistical Bioinformatics, Hasselt University, Diepenbeek, Belgium; 3 Department of Parasitology, College of Veterinary Medicine and Agriculture, Addis Ababa University, Bishoftu, Ethiopia; 4 Department of Veterinary Microbiology and Parasitology, College of Veterinary Medicine and Biomedical Sciences, Sokoine University of Agriculture, 3019, Morogoro, Tanzania; 5 Department of Bio-molecular Resources and Bio-Laboratory Sciences (BBS), College of Veterinary Medicine, Makerere University, Kampala, Uganda; 6 Department of Veterinary Tropical Diseases, Faculty of Veterinary Science, University of Pretoria, Onderstepoort, South Africa; 7 National Institute of Agricultural Research (INRAB), Zootechnical, Veterinary and Halieutic Research Laboratory (LRZVH), 01 BP 884 Cotonou, Benin; 8 Université Nangui Abrogoua, UFR Sciences de la Nature, 02 Bp 801, Abidjan 02, Côte d’Ivoire; 9 Department of Animal Biology and Conservation Science, School of Biological Sciences, College of Basic and Applied Sciences, University of Ghana, Legon-Accra, Ghana; 10 National Veterinary Research Institute, Vom, Plateau State Nigeria; 11 Bill & Melinda Gates Foundation, Seattle, WA USA; 12 Clinvet International Pty (Ltd), 1479 Talmadge Hill South, Waverly, NY 14892 USA; 13 Clinglobal, B03/04, The Tamarin Commercial Hub, Jacaranda Avenue, Tamarin, 90903 Mauritius; 14 Research Center for Tropical Diseases and Vector Control (RTC), Department of Veterinary Pharmacy, Clinics and Comparative Medicine, School of Veterinary Medicine and Animal Resources, College of Veterinary Medicine, Animal Resources and Biosecurity, Makerere University, Kampala, Uganda

**Keywords:** *Amblyomma variegatum*, *Rhipicephalus microplus*, *Babesia bovis*, *Rhipicephalus appendiculatus*, Sub-sahara Africa

## Abstract

**Background:**

The majority of the African population lives in rural areas and depends on agriculture for their livelihoods. To increase the productivity and sustainability of their farms, they need access to affordable yield-enhancing inputs of which parasite control is of paramount importance. We therefore determined the status of current tick species with the highest economic impact on cattle by sampling representative numbers of animals in each of seven sub-Saharan countries.

**Methods:**

Data included tick species’ half-body counts from approximately 120 cattle at each of two districts per country, collected four times in approximately 1 year (to include seasonality). Study sites were chosen in each country to include high cattle density and tick burden.

**Results:**

East Africa (Ethiopia, Uganda and Tanzania) showed overall a higher diversity and prevalence in tick infestations compared to West African countries (Benin, Burkina Faso, Ghana and Nigeria). In East Africa, *Amblyomma variegatum* (vector of *Ehrlichia ruminantium*), *Rhipicephalus microplus* (*Babesia bovis, B. bigemina, Anaplasma marginale*), *R. evertsi evertsi* (*A. marginale*) and *R. appendiculatus* (*Theileria parva*) were the most prevalent tick species of economic importance. While the latter species was absent in West Africa, here both *A. variegatum* and *R. microplus* occurred in high numbers. *Rhipicephalus microplus* had spread to Uganda, infesting half of the cattle sampled. *Rhipicephalus microplus* is known for its invasive behaviour and displacement of other blue tick species, as observed in other East and West African countries. Individual cattle with higher body weights, as well as males, were more likely to be infested. For six tick species, we found reduced infestation levels when hosts were treated with anti-parasiticides.

**Conclusions:**

These baseline data allow the determination of possible changes in presence and prevalence of ticks in each of the countries targeted, which is of importance in the light of human-caused climate and habitat alterations or anthropogenic activities. As many of the ticks in this study are vectors of important pathogens, but also, as cattle may act as end hosts for ticks of importance to human health, our study will help a wide range of stakeholders to provide recommendations for tick infestation surveillance and prevention.

**Graphical abstract:**

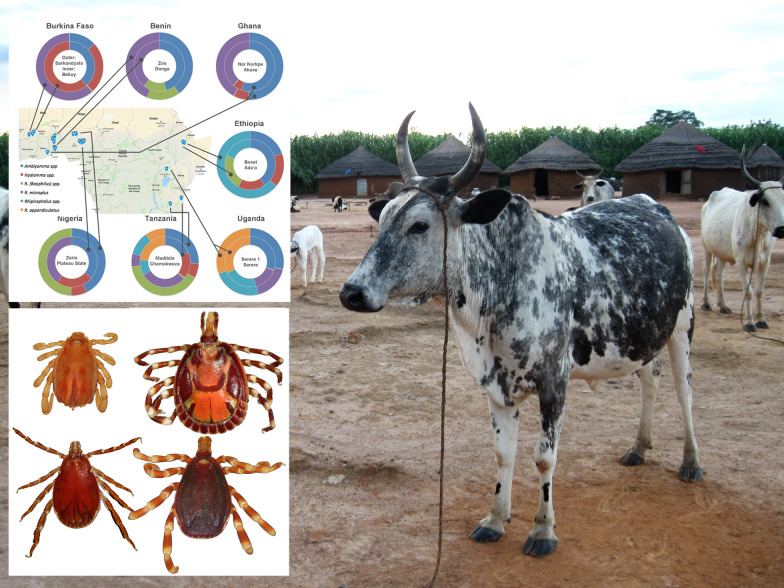

**Supplementary Information:**

The online version contains supplementary material available at 10.1186/s13071-023-05801-5.

## Background

The majority of the human African population lives in rural areas where they heavily rely on agriculture, including livestock production [1]. Often sub-Saharan farmers belong to resource-constrained farming communities and struggle to maintain minimal life standards, not seldomly because of the harm caused by ecto- and endoparasites, including (invasive) vector-borne infectious diseases [2]. Affordable yield-enhancing inputs are needed to increase productivity and sustainability of their farms, to which they need easy access. For successful parasite control an essential prerequisite is standardised surveys of the current state of tick exposures and tick-borne haemoparasite transmission outcomes. Timely surveys provide opportunities to private and governmental institutions to collaborate and invest in tick infestation control measures. Such information is not only needed for advocacy to guide national and development partner budget allocation and support, but also serves the pharmaceutical industry to justify development and deployment of new and effective compounds for local tick infestation control. Furthermore, these baseline data are an essential requirement for the identification and measurement of effective changes in burdens of ticks and the pathogens they vector after anti-parasite interventions. However, because of the current lack of standardised and affordable diagnostics in many African countries, it is so far not possible to estimate the real burden linked to ticks, risk and associated economic damage.

As part of a parasite mapping project which focused on enhancing livestock care across Africa, we initiated a standardised multi-country surveillance study to assess the current status of important endemic and invasive ticks of cattle across seven sub-Saharan African countries (West Africa: Burkina Faso, Ghana, Benin, Nigeria and East Africa: Ethiopia, Uganda, Tanzania) [3–5]. Our veterinary network was able to simultaneously sample representative numbers of cattle (within farms) and consider the individual variation in tick infestations, and this over a time window of approximately 1 year. In Africa, ticks constitute a major constraint to livestock production for most small-scale livestock farmers, even though smallholders and pastoralists may not easily detect the effects of those ectoparasites on their animals. The constraints to livestock production posed by tick infestation are either direct or indirect. Direct constraints include, but are not limited to, reduced weight gains, lower growth rate, reduced nutrient utilisation, lower meat and milk yield, reduced value of hides, tick paralysis and the relief of individual animals suffering by culling. Indirect constraints include transmission of some of the most important livestock diseases: anaplasmosis, babesiosis, theileriosis (East Coast fever) and heartwater [1, 6].

Often the vector status of ticks is unknown and/or has not been experimentally assessed, which is why we aimed to document the spatio-termporal varation in as many tick species as possible, although the main focus of this work was on the most common ticks of known socio-economic importance given their known vector competence for important disease-causing micro-parasites [6]: (1) *Amblyomma gemma* and *variegatum*, which are considered the main vectors for *Ehrlichia ruminantium* [7], the causal agent of heartwater—an obligate intracellular bacterium invading endothelial cells in cattle, sheep, goats and wild ruminants with a frequently fatal outcome. (2) A suite of tick species (*Hyalomma rufipes*, *Rhipicephalus decoloratus*, *R. pulchellus*, *R. microplus*, *R. evertsi evertsi* and *R. annulatus*) successfully transmits *Anaplasma marginale*, the etiological agent of bovine anaplasmosis. In animals with severe degree of infection, symptoms are characterised by anemia, weight loss and often death [8]. (3) *Rhipicephalus microplus* and *R. annulatus* are known to successfully transmit *Babesia bovis*, an intra-erythrocytic protozoan causing bovine babesiosis. Another less virulent and more widespread *Babesia* species is *B. bigemina* [9], which is transmitted in addition to the latter two tick species, by *R. decoloratus*. (4) *Rhipicephalus appendiculatus* is the main vector of *Theileria parva*, causing East Coast fever [10]. Besides erythrocytes, this protozoa also affects endothelial and white blood cells and causes a fatal disease in the most susceptible animals, especially calves [6, 11, 12].

The current distribution of the most economically important ticks parasitising cattle is still uncertain especially for the cattle tick *R. microplus*, the vector of bovine babesiosis. After its introduction in West Africa in 2004–2007, the tick spread efficiently in the West African region and displaced the local one-host ticks [13–16]. A further spread was noticed to Nigeria in 2014 [17] and to Cameroon in 2016 [18]. At the same time, *R. microplus* expanded its distribution in East Africa to Angola and Uganda and displaced *R. decoloratus* in several countries like Tanzania [4, 19–21]. The distribution of the other important cattle ticks like *A. variegatum* and *R. appendiculatus* is more stable and no significant distribution changes or introductions have been published. *Amblyomma variegatum* is by far the most distributed species whereas *R. appendiculatus* is restricted to eastern and southern African countries except in those areas that are too dry where it is replaced by *Rhipicephalus zambeziensis* [22–24]. Implementation of effective measures to control ticks and hence the pathogens they vector (i.e. establishment of proper treatment strategies and prevention) relies on the elucidation of local tick life cycles, including: the level of susceptibility of organisms on which ticks potentially feed as well as tick exposure risks in the community of potential hosts. Aside from an updated cross-sectional tick surveillance, we therefore explored associations between the animal’s half-body infestation status and exposure risk factors (intrinsic as well as extrinsic) with the main focus on ticks of high economic impact to livestock in smallholder livestock production systems. In summary, in this article the following questions are addressed: (1) to what extent does species composition on livestock vary macro-geographically, (2) which general host characteristics (sex, condition and body weight) are associated with tick infestations and (3) are there indications that current anti-parasite treatments have an effect on tick infestation success? Outcomes and documentation will inform the process of establishing effective and sustainable control programmes for the benefit of small-scale rural livestock farmers and inspire current and future intervention plans.

## Methods

### Study design and sites

Cattle were included in this survey, irrespective of gender, if they were 1 to 2 years old, had not been treated with a topical or systemic acaricide during the 2 weeks prior to the sampling visit and were not excessively fractious in that they posed a danger to themselves or study site personnel. Cattle were not identified individually. Ideally, ticks were collected from 120 cattle during every sampling at each site. The number of herds sampled at each site varied but at least five animals per herd were sampled.

**Fig. 1:**
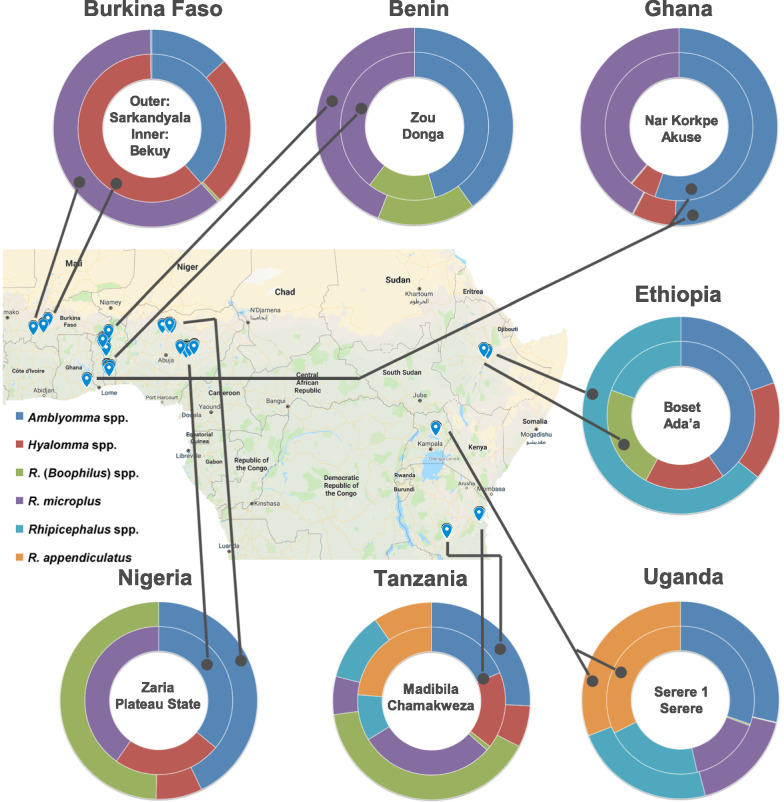
Overview of the sampling locations in seven African countries. Each section in a donut represents the proportion of cattle individuals infested with a particular taxon

In each country, two districts **(**Fig. 1) were selected with known high cattle density, hence with expected high prevalence of ticks and tick-borne diseases.

Two sites sampled are localised in the South West Burkina Faso: Sarkandiala in the province of La Léraba and Bekuy in the province of Houet. The climate in this most western region of the country is characterised by average minimum and maximum temperatures of ± 22 and 34 °C with an annual precipitation of ± 1000 mm. On average two-thirds of the cattle population follows a husbandry system of communal grazing whereas one-third small or large transhumance.

Ghana: Akuse and Narh Korkpe are both located in the Lower Manya Krobo district. Both communities are located around the southern banks of the Volta Lake. The temperature is between 24 and 31 °C with annual precipitation of ± 800 mm. Half of the cattle population sampled is sedentary whereas the other half is part of the transhumance.

Benin: Donga Department and Zou Department, Donga in the mid-west of the country and Zou in the south. The climate in Zou is between 24 and 33 °C with a precipitation of around 1150 mm whereas Donga further north has a temperature range between 22 and 34 °C with annual precipitation of ± 900 mm. Most animals sampled had communal grazing.

Nigeria: Plateau State is located in the central part of Nigeria with an average temperature between 22 and 34 °C and annual precipitation of ± 1500 mm. Kaduna State (Zaria) has a warmer climate with average temperatures between 22 and 36 °C and annual precipitation of about 1100 mm. Most animals were classified under communal grazing although about one fourth was part of transhumance.

Ethiopia: Typical representative sites in central and eastern Oromia; Ada'a is located in the central Ethiopian highland with average temperatures between 12 and 26 °C and annual precipitation of ± 1500 mm. Boset is situated in the lowlands of Ethiopia at an average temperature between 11 and 24 °C and precipitation of ± 1200 mm. All of the animals were classified under communal grazing systems.

Uganda: The two sampling sites were located in Serere district, east of Lake Kyoga. The area has an average temperature between 19 and 31 °C with precipitation of ± 3200 mm. No transhumance was observed among the animals sampled in this region.

Tanzania: Chamakweza in Bagamoyo district (Coast region) and Madibila (Mbarali district; Mbeya region) were sampled. Temperature in Chamakweza ranges between 20 and 32 °C and annual rainfall is around 840 mm. In Madibila, close to the north of Lake Malawi, temperature ranges between 18 and 29 °C with a precipitation of ± 650 mm. Sampling sites consisted of smallholder livestock farmer settlements with mainly sedentary cattle herds located in the selected localities.

The survey was conducted over a period of 1 year (August 2016 to June 2017). Fieldwork consisted of four quaternary sampling visits. The first sampling took place in August 2016, the second sampling took place in November 2016, the third sampling was in April 2017 and the fourth sampling in June 2017 to cover all the seasons in the different countries. Individual sampling duration for each visit (to both sites) was approximately 2 weeks (approximately 1 week per site) but varied depending on logistical challenges encountered. During each visit, the target was to sample 240 cattle per country (approximately 120 per site). Ticks were collected from 7070 animals, roughly 240 animals per visit per district.

### Assesment of intrinsic and extrinsic ecological risk factors

The weight of cattle (sampled for parasites from small holder herds) was estimated using a RONDO^®^ tape (Kyron Labs, Johannesburg, South Africa) according to the manufacturer’s recommendations. Body condition scoring was conducted for these animals according to work instructions provided in the protocol (see Additional File 2: Protocol). In addition, we determined gender, the type of husbandry and number of ticks. Blood samples were taken for molecular screening of the tick-borne haemoparasites [3]. For anti-parasiticides (acaricides and anthelmintics), however, the product type was often not or incompletely registered, or was registered for each of the cattle individually as well as the time since last treatment.

### Tick collection and identification

Ticks collected from each animal were stored in plastic vials containing 70% ethanol. The vials were then transported to local laboratories for identification of the ticks. In brief, half-body sampling was performed on five predilection sites: (i) the inner and outer forelegs, hind legs and abdomen; (ii) tail and anal area; (iii) head and neck; (iv) lateral area and dorsal area from shoulders to tail base; (v) ears. The ticks were removed using forceps. The collection was performed for about 15 min in total from all the predilection sites. For heavily infested animals, the ticks remaining on the animal after the 15-min collection period were counted and recorded. The different genera were recorded separately. Ticks were identified based on morphology using a stereoscope (80-fold magnification). For better visualising the hypostome dentition of ticks belonging to the subgenus *Rhipicephalus* (Boophilus), a microscope (100- to 200-fold magnification) was used. Only adult specimens were identified to species level using both taxonomic descriptions [24] and morphological keys [25, 26]. The identification and confirmation of *R. microplus* in areas where the tick had not been found before were done molecularly as described by Muhanguzi and collaborators [3].

### Statistical analysis

The data possess a hierarchical structure with tick species infestation levels (binary: 0/1, loads: [0− ∞]) for each host individual nested within a farm and farm nested within a district. To obtain valid statistical inferences, the dependence structure needed to be considered. For these purposes, generalised estimation equation models were fitted onto the data (see [3]) considering the statistical dependence of observations within farms per visit (nested within districts) by adding an exchangeable working correlations at the sublevel (i.e. farm/visit) [27]. The residuals for infestation proportions were assumed to follow a binomial distribution (logit-link), while residuals of infestation loads followed negative binomial distributions (log-link). The proportions (i.e. prevalences) and loads were included as response variables in models with the following explanatory variables: the individual’s intrinsic (age, sex, body condition, de-worming drugs and/or ectoparasiticide) and extrinsic risk factors (husbandry: communal vs. transhumance). For each of the continuous explanatory variable (i.e. tick load, body weight and body condition score), we mean-centred the data at farm and district level because of substantial geographic variation (Table 1). Doing so, outcomes allowed us to differentiate at which level (i.e. individual, farm, district level) and how the variation in pathogen prevalence was explained. Our emphasis was to determine associations found at lower levels (i.e. individual and farm) as these are less confounded by ecological biases. We considered generalised continent-wise comparison among the four visits to be of little epidemiological relevance, given that for each country climatic seasonality differs. Therefore, the investigation of differences between visits was restricted within the district by adding the temporal macro-geographic variation as nested random effect within the district.

**Table 1:** Macro-geographic variation in the continuous and categorical variables included in the generalised estimation equations

	Burkina Faso (no. farms: 19)		Ghana (7)		Benin (19)		Nigeria (7)		Ethiopia (24)		Uganda (89)		Tanzania (10)	
Condition female	4.83 ± 1.79		5.43 ± 0.41		6.17 ± 0.4		4.46 ± 0.36		4.74 ± 1.01		5.93 ± 1.18		5.37 ± 0.67	
Male	5.03 ± 1.78		5.29 ± 0.49		6.11 ± 0.41		4.39 ± 0.31		5.06 ± 0.95		5.88 ± 0.79		5.49 ± 0.78	
Body weight female	182.85 ± 37.31		93.95 ± 20.04		151.9 ± 40.99		135.16 ± 17.56		122.02 ± 21.8		203.88 ± 51.03		126.03 ± 27.22	
Male	186.21 ± 44.31		95.32 ± 21.18		152.31 ± 42.22		121.47 ± 22.74		130.4 ± 23.53		227.64 ± 58.1		119.86 ± 22.68	
Proportion females (over total)	43.4 ± 20.2		44.9 ± 12.9		50.9 ± 13.3		32.4 ± 20.1		51.1 ± 23.5		54 ± 34		46.7 ± 18.2	
Proportion transhumance (over total)	15.7 ± 36.4		54.5 ± 50.4		0 ± 0		24.2 ± 42.8		0 ± 0		0 ± 0		0 ± 0	
Proportion treated with anti-parasitic drugs	27.7 ± 44.2		43.9 ± 42.7		65.1 ± 46.9		8.4 ± 26.1		1.9 ± 10.6		0.3 ± 4.2		3 ± 7.2	
	*C*	27.6 ± 44.2	*C*	0.0 ± 0.0			*C*	11.0 ± 29.4						
	*T*	26.1 ± 44.9	*T*	80.5 ± 18.2			*T*	0.0 ± 0.0						

Note: Data are presented as averages at farm-visit level ± 1 standard deviation

Husbandry: *T* transhumance, *C* communal

For all analyses, a stepwise backward selection procedure was used to select the best model. At each step, we excluded the fixed factor with the highest non-significant *P*-value (*P* > 0.05), re-ran the model and examined the *P*-values of the fixed factors in the reduced model. Model reduction continued until only significant factors (*P* < 0.05) and their lower order interaction terms were left [28]. As part of the description of parasite communities, the Shannon diversity index was computed [29]. All data management and statistical analyses were done in SAS v 9.3 (SAS Institute, Cary, NC, USA).

## Results

### Tick species collected

Ticks were identified up to species level. Seventeen tick species of three genera were identified. One species was recorded in all seven countries: *A. variegatum*. All four countries of West Africa had records of *R. microplus*. All three countries in East Africa had records of *R. decoloratus* and *R. evertsi evertsi*. The distribution of ticks among the cattle individuals within a farm was heavily skewed, based on the estimated shape parameters that characterise negative binomial distributions (all *K* < 1.77; Tables 2, 3) except for *R. appendiculatus* in Uganda (*K* = 7.1; prevalence 98.05%; tick loads 162.78 ± 92.5 per animal).

**Table 2:** Tick prevalence, infestation loads and species diversity in West African countries

	*West*	*Burkina Faso*	*K*	*Ghana*	*K*	*Benin*	*K*	*Nigeria*	*K*
*Amblyomma*									
*A. variegatum*	37.60 (12.79 ± 33.47)	17.54^a^ (0.62 ± 1.77^a^)	0.01(1)	56.06^b^ (15.04 ± 26.1^b^)	0.49(38)	50.88^b^ (31.32 ± 53.79^c^)	0.43(28)	24.66^a^ (1.95 ± 4.92^d^)	
*A. gemma*	0.00 (0 ± 0)	0.00 (0 ± 0)		0.00 (0 ± 0)		0.00 (0 ± 0)		0.00 (0 ± 0)	
*Hyalomma*									
*H. rufipes*	7.53 (0.5 ± 2.31)	23.32 (1.26 ± 3.03)	0.58(21)	6.76 (0.73 ± 3.35)	0.06(15)	0.00 (0 ± 0)		0.00 (0 ± 0)	
*H. truncatum*	4.15 (0.22 ± 1.37)	5.88 (0.18 ± 0.98)	0.18(8)	0.00 (0 ± 0)		0.00 (0 ± 0)		11.46 (0.77 ± 2.57)	
*H. impressum*	0.12 (0 ± 0.06)	0.47 (0.01 ± 0.11)	0.07(1)	0.00 (0 ± 0)		0.00 (0 ± 0)		0.00 (0 ± 0)	
*H. albiparmatum*	0.02 (0 ± 0.29)	0.00 (0 ± 0)		0.10 (0.02 ± 0.6)		0.00 (0 ± 0)		0.00 (0 ± 0)	
*Rhipicephalus*									
*R. microplus*	34.40 (18.6 ± 61.49)	25.88^a^ (1.55 ± 3.67^a^)	0.36(4)	43.24^b^ (7.03 ± 15.44^b^)	0.4(37)	49.74^c^ (58.27 ± 106.56^c^)	0.51(28)	16.51^d^ (2.61 ± 10.71^a^)	
*R (Boophilus*) spp.	4.82 (2.8 ± 19.16)	0.19 (0 ± 0.04)		0.10 (0 ± 0.03)		17.40 (10.26 ± 35.62)	0.14(16)	0.00 (0 ± 0)	
*R. decoloratus*	2.69 (0.43 ± 5.21)	0.00 (0 ± 0)		0.10 (0.02 ± 0.71)		0.00 (0 ± 0)		11.46 (1.85 ± 10.67)	
*R. annulatus*	0.24 (0.11 ± 3.48)	0.00 (0 ± 0)		0.00 (0 ± 0)		0.88 (0.39 ± 6.66)	0.02(2)	0.00 (0 ± 0)	
*R. geigyi*	0.02 (0 ± 0.14)	0.00 (0 ± 0)		0.00 (0 ± 0)		0.09 (0.01 ± 0.26)		0.00 (0 ± 0)	
*R. sanguineus*	0.02 (0 ± 0.05)	0.09 (0 ± 0.09)		0.00 (0 ± 0)		0.00 (0 ± 0)		0.00 (0 ± 0)	
*R. appendiculatus*	0.00 (0 ± 0)	0.00 (0 ± 0)		0.00 (0 ± 0)		0.00 (0 ± 0)		0.00 (0 ± 0)	
*R. lunulatus*	0.00 (0 ± 0)	0.00 (0 ± 0)		0.00 (0 ± 0)		0.00 (0 ± 0)		0.00 (0 ± 0)	
*R. evertsi evertsi*	0.00 (0 ± 0)	0.00 (0 ± 0)		0.00 (0 ± 0)		0.00 (0 ± 0)		0.00 (0 ± 0)	
*R. praetextatus*	0.00 (0 ± 0)	0.00 (0 ± 0)		0.00 (0 ± 0)		0.00 (0 ± 0)		0.00 (0 ± 0)	
*R. pravus*	0.00 (0 ± 0)	0.00 (0 ± 0)		0.00 (0 ± 0)		0.00 (0 ± 0)		0.00 (0 ± 0)	
*R. pulchellus*	0.00 (0 ± 0)	0.00 (0 ± 0)		0.00 (0 ± 0)		0.00 (0 ± 0)		0.00 (0 ± 0)	
No. screened	4168	1055		1006		1138		969	
No. infested (%)	2698 (64.73)	634 (60.09)		691 (68.68)		896 (78.73)		477 (49.22)	
Co-infestation	39.04	19.25		50.59		50.88		26.85	
Shannon’ index		1.33		0.93		1.05		1.04	

Prevalence (%) and average tick load (number of ticks/individual ± standard error)

K: Negative binomial shape parameter (the higher, the less skewed distributions)—calculated for farm × time combinations with > 10 animals sampled and a tick prevalence > 5%. Criteria were not met in Nigeria. For taxa with overall regional prevalence ≥ 10%: the same letters indicate that the contrast between countries is not statistically different from zero

**Table 3:** Tick prevalence, infestation loads and species diversity in East African countries

	*East*	*Ethiopia*	*K*	*Uganda*	*K*	*Tanzania*	*K*
*Amblyomma*							
*A. variegatum*	50.57 (13.86 ± 28.11)	19.98^a^ (8.88 ± 26.67^a^)	0.29 (13)	91.38^b^ (27.42 ± 34.42^b^)	1.76 (25)	39.81^c^ (5.15 ± 13.64^a^)	0.47 (33)
* A. gemma*	3.58 (0.56 ± 5.1)	10.61 (1.67 ± 8.76)	0.1 (9)	0.00 (0 ± 0)		0.21 (0.01 ± 0.28)	
*Hyalomma*							
*H. rufipes*	9.89 (1 ± 4.76)	7.49^a^ (0.73 ± 3.98^a^)	0.08 (10)	0.00 (0 ± 0)		22.23^b^ (2.28 ± 7.05^b^)	0.19 (32)
*H. truncatum*	3.51 (0.37 ± 2.42)	10.09 (1.06 ± 4.05)		0.00 (0 ± 0)		0.52 (0.04 ± 0.75)	0.01 (1)
*H. albiparmatum*	0.38 (0.02 ± 0.31)	0.00 (0 ± 0)		0.00 (0 ± 0)		1.14 (0.05 ± 0.54)	0.05 (3)
*H. impressum*	0.00 (0 ± 0)	0.00 (0 ± 0)		0.00 (0 ± 0)		0.00 (0 ± 0)	
*Rhipicephalus*							
*R. appendiculatus*	44.16 (55.81 ± 93.17)	0.00^a^ (0 ± 0^a^)		98.05^b^ (162.78 ± 92.5^b^)	7.1 (25)	33.71^c^ (3.43 ± 7.42^c^)	0.5 (32)
*R. evertsi evertsi*	32.72 (3.84 ± 9.35)	9.05^a^ (0.59 ± 2.41^a^)	0.08 (13)	69.03^b^ (9.44 ± 13.79^b^)	1.58 (25)	19.65^c^ (1.42 ± 4.15^c^)	0.15 (34)
*R. microplus*	29.83 (10.12 ± 34.59)	0.00^a^ (0 ± 0^a^)		51.08^b^ (7.94 ± 18.65^b^)	0.46 (25)	38.06^c^ (22.37 ± 54.66^c^)	0.54 (32)
*R. decoloratus*	13.85 (1.94 ± 9.21)	10.93^a^ (1.6 ± 10.06^a^)	0.17 (12)	0.72^b^ (0.03 ± 0.33^b^)	0.05 (3)	29.99^c^ (4.21 ± 12.05^c^)	0.34 (23)
*R. pulchellus*	10.02 (3.52 ± 15.96)	30.28^a^ (10.63 ± 26.35^a^)	0.9 (10)	0.00^b^ (0 ± 0^b^)		0.00^c^ (0 ± 0^c^)	
*R. praetextatus*	1.17 (0.12 ± 1.57)	3.54 (0.37 ± 2.72)	0.05 (4)	0.00 (0 ± 0)		0.00 (0 ± 0)	
*R. lunulatus*	0.93 (0.1 ± 1.67)	2.81 (0.31 ± 2.89)	0.11 (5)	0.00 (0 ± 0)		0.00 (0 ± 0)	
*R (Boophilus) *spp.	0.14 (0.01 ± 0.45)	0.00 (0 ± 0)		0.00 (0 ± 0)		0.41 (0.04 ± 0.77)	0.01 (1)
*R. pravus*	0.03 (0.0 ± 0.05)	0.10 (0 ± 0.08)		0.00 (0 ± 0)		0.00 (0 ± 0)	
*R. annulatus*	0.00 (0 ± 0)	0.00 (0 ± 0)		0.00 (0 ± 0)		0.00 (0 ± 0)	
*R. geigyi*	0.00 (0 ± 0)	0.00 (0 ± 0)		0.00 (0 ± 0)		0.00 (0 ± 0)	
*R. sanguineus*	0.00 (0 ± 0)	0.00 (0 ± 0)		0.00 (0 ± 0)		0.00 (0 ± 0)	
No. screened	2903	961		975		967	
No. infested (%)	2311 (79.60)	564 (58.68)		961 (98.56)		786 (81.28)	
Co-infestation	77.66	54.09		97.7		70.11	
Shannon’ index		1.99		1.37		1.82	

Note: Prevalence (%) and average tick load (number of ticks/individual ± standard error)

K: Negative binomial shape parameter (the higher, the less skewed)—calculated for farm *×* time combinations with > 10 animals sampled and a tick prevalence > 5%. For taxa with overall prevalence ≥ 10%: the same letters indicate that the contrast between countries is not statistically different from zero

### Macro-geographic variation in tick prevalences

**Fig. 2:**
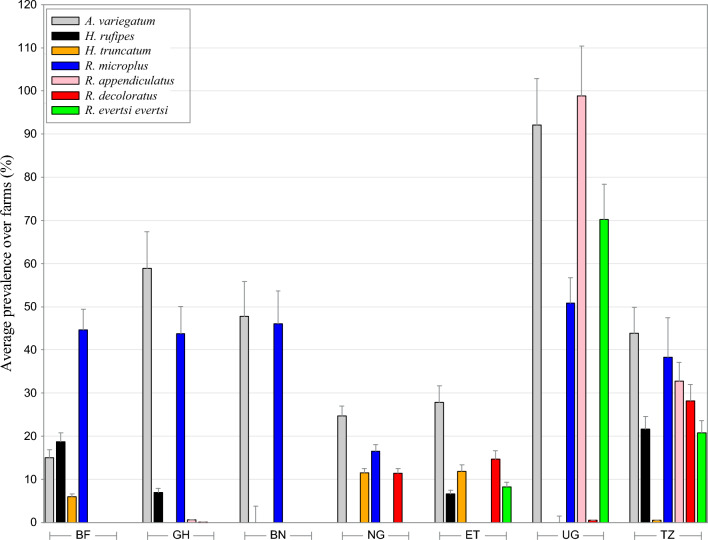
Macro-geographic variation in tick prevalence. Overall averages (+ 1 standard deviation) are calculated over the different farms (nested within the district). *BF* Burkina Faso, *GH* Ghana, *BN* Benin, *NG* Nigeria, *ET* Ethiopia, *UG* Uganda, *TZ* Tanzania

Countries differed from each other when considering the animal’s tick prevalences (Table 2, 3 and Fig. 2 for macro-geographic overview of most prevalent tick species), and for most tick-country combinations also strong temporal variation was observed (Additional file 1: Tables S1, S2). *Amblyomma variegatum* was by far the most prevalent tick in both West (37.60%) and East Africa (50.57%), with Uganda showing the highest prevalence (91.38%), but with strong differences among countries in prevalences (*χ*2 = 1832.57; df = 6; *P *< 0.0001) and loads (*χ*2 = 1323.78; df = 6; *P* < 0.0001). The congeneric *A. gemma* was almost absent throughout the continent (range: 0.00–10.67%). *Hyalomma rufipes* was not found in Uganda, Nigeria and Benin, but prevalences varied between 6.76% (Ghana) and 23.32% (Burkina faso) in all other countries. In the *Rhipicephalus* genus (12 species identified) strong contrasts between West and East Africa were observed: Several species that were commonly found in East African cattle were (almost) absent in West Africa (*R. appendiculatus*, *R. evertsi*, *R. pulchellus*), one of the the reasons why West African countries had a lower Shannon’s diversity index (range: 0.93–1.33) than the East African countries (range: 1.37–1.99). Highest species’ diversity index was obtained from Ethiopian data (Shannon’s H: 1.99), where no fewer than eight different *Rhipicephalus* species were identified. Uganda showed the highest prevalences of *R. appendiculatus* (98.05%) and *R. evertsi* (69.03%) infested cattle. The invasive *R. microplus* was commonly found in West Africa (range: 16.51–49.74%; *χ*2 = 994.25; df = 3; *P* < 0.0001) and East Africa (38.06–51.08%%; *χ*2 = 907.00; df = 2; *P* < 0.0001), but was not observed in Ethiopia. In West Africa, *R. decoloratus* was mainly found in Nigerian cattle (11.46%) and was nearly absent in other two countries. For East Africa, in the latter tick species as well as in *R. appendiculatus* strong variation was found with absences in Uganda and Ethiopia of *R. decoloratus* and *appendiculatus*, respectively. For *R. microplus*, cattle kept in communal husbandry (Burkina Fasso: 40.96%; Ghana: 63.76%) had significantly fewer ticks than those kept in transhumance settings in the same districts (Burkina Faso: 5.24%; Ghana: 34.80%). The reverse pattern however was found in Nigeria (communal: 16.32% vs. transhumance: 63.68%). Though almost absent throughout the continent, *R. pulchellus* (30.28%) and *H. truncatum* (10.09%) were relatively common in Ethiopia.

### Ecological correlations

The farm variation in ecological risk factors for each of the countries is presented in Table 1. Female cattle kept in communal husbandry settings showed lower tick loads than males (Table 4a) in *R. microplus*, *A. variegatum*, *R. appendiculatus* and *R. decoloratus* (log_female-male_ < − 0.14 ± 0.06 ticks; *Z* < − 2.21; *P* < 0.026). In the subset of cattle kept in transhumance settings the same pattern was observed in *R. microplus* (log_female-male_: − 0.63 ± 0.17 ticks; *Z* < − 3.68; *P* < 0.001) and *A. variegatum* (log_female-male_: − 0.22 ± 0.08 ticks; *Z* < − 2.79; *P* = 0.005). In contrast to the burdens (i.e. loads), no sex differences were found in tick prevalence, except for *R. decoloratus* (communal; logit_female- male_ = − 0.15 ± 0.06; *Z* = − 2.51; *P* = 0.012).

For all tick species, tick loads showed a positive association with body weight (a proxy for the age and body surface) (communal, range effect sizes, log: 3.17 10^–4^–74.66 10^–4^ ticks/kg; Table 4; transhumance, range effect sizes, log: 25.7 10^–4^–38.6 10^–4^ ticks/kg; Table 5). One exception to this rule were patterns of infestation in *R. microplus* (Ghana only) of transhumance settings, where the heavier animals had fewer ticks (log: − 14.0 ± 5.2 10^–3^ ticks/kg; *Z* = − 2.70; *P* = 0.007). Effect sizes of the estimates in tick prevalences followed the same directions as those for tick loads however were less often statistically significant (in *H. rufipes*, *R. appendiculatus*, *R. microplus* of communal settings; in *H. rufipes* of transhumance settings).

**Table 4:** Communal husbandry: parameter estimates (± empirical standard error) from ecological models

		*R.microplus * BF, Gh, Be, Ug, Ta	*R. decoloratus * Ni, Et, Ta	*R. evertsi evertsi * Et, Ug, Ta	*R.appendiculatus* Ug, Ta	*H. truncatum* Ni, Et	*H. rufipes * GH, Ta,BF	*A. variegatum * All countries	*A. gemma * Et
Covariate									
Weight (age proxy)	Prev	0.80 ± 0.71 10^–4^ ns	3.63 ± 1.43 10^–4^ ns	**2.47 ± 1.07 10**^–4^*	22.3 ± 15 10^–4^ ns	**48.2 ± 18.5 10**^–4^**	25.3 ± 14.3 10^–4^ ns	**33.4 ± 0.6 10**^–4^***	12.1 ± 0.4**
	Load	**3.17 ± 0.78 10**^–4^*	3.73 ± 2.22 10^–4^ ns	**3.73 ± 0.93 10**^–4^***	**16.4 ± 5.3 10**^–4^**	**74.6 ± 23.0 10**^–4^**	**28.6 ± 14.4 10**^–4^*	**38.5 ± 0.6 10**^–4^***	15.3 ± 0.3***
Sex									
Female vs. male	Prev	− 0.11 ± 0.06 ns	− 0.15 ± 0.06*	0.18 ± 0.11 ns	− 0.23 ± 0.13 ns	0.07 ± 0.15 ns	0.10 ± 0.13 ns	− 0.04 ± 0.06 ns	0.24 ± 0.28 ns
	Load	− 0.14 ± 0.06*	− 0.24 ± 0.11*	0.08 ± 0.11 ns	− 0.18 ± 0.07*	0.24 ± 0.16 ns	0.06 ± 0.13 ns	− 0.15 ± 0.07*	0.46 ± 0.52 ns
Body condition	Prev	0 ± 0.04 ns	0.05 ± 0.04 ns	− 0.04 ± 0.05 ns	− 0.05 ± 0.07 ns	0.01 ± 0.08 ns	− 0.07 ± 0.07 ns	0.01 ± 0.03 ns	− 0.34 ± 0.09***
	Load	− 0.05 ± 0.04 ns	0.09 ± 0.07 ns	− 0.07 ± 0.05 ns	− 0.04 ± 0.04 ns	− 0.02 ± 0.11 ns	− 0.13 ± 0.05**	− 0.05 ± 0.04 ns	− 0.72 ± 0.23**
Parasiticide		BF	Ni			Ni	BF	BF, Be, Ni	
< 1 month vs. never	Prev	0.35 ± 0.33 ns	NC			NC	− 0.92 ± 0.55 ns	− 0.05 ± 0.34 ns	
	Load	− 0.47 ± 0.24*	NC			NC	− 0.55 ± 0.44 ns	0.17 ± 0.14 ns	
1–2 months vs. never	Prev	NA	NC			NC	NA	− 0.47 ± 0.13***	
	Load	NA	NC			NC	NA	− 27.10 ± 4.52***	
> 2 months vs. never	Prev	0.04 ± 0.41 ns	NC			NC	− 1.20 ± 0.64*	− 0.54 ± 0.54 ns	
	Load	− 0.52 ± 0.37 ns	NC			NC	− 1.53 ± 0.73*	− 0.11 ± 0.19 ns	
Treated vs. never vs. never	Prev	− 0.25 ± 0.34 ns	− 0.58 ± 0.39 ns			− 1.47 ± 1.70 ns	− 0.42 ± 0.31 ns	− 0.21 ± 0.22 ns	
	Load	− 0.74 ± 0.25**	− 0.98 ± 0.21 ***			− 1.41 ± 0.21 ns	− 0.41 ± 0.28 ns	− 0.15 ± 0.07*	

Numbers in bold are linked to 'statistically significant' effects

Note: Generalised estimation equations that model the tick species’ prevalence (levels: 0, 1; ‘Pre’) and infestation loads (‘Lo’) in cattle of smallholder rural areas. For a given taxon, only countries with a prevalence of at least 10% were included. Individual body weight and body condition were mean-centered at farm level (nested within country districts). Effects of parasiticides were tested only in Burkina Faso (27.6% of animals treated), Benin (65.1%) and Nigeria (11.0%). Prevalence: model estimates reflect the probability that tick has level ‘1’. Load: model estimates reflect the associations between number of ticks and a unit increase in explanatory variable. Insufficient farm-visit combinations in which more than one treatment condition has been applied. No significant associations were found in *R. pulchellus*

*Bf* Burkina, *Gh* Ghana, *Be* Benin, *Ni* Nigeria, *Et* Ethiopia, *Ug* Uganda, *Ta* Tanzania. *NA* not applicable, since no data available, *NA* not applicable, since no data available, *NC* no model convergence

***P < 0.001

**P < 0.01

*P  < 0.05

*ns P* > 0.05

**Table 5:** Transhumance husbandry: Parameter estimates (± empirical standard error) from ecological models

		*R. microplus* Gh, Ni	*H. truncatum* Ni	*H. rufipes* BF	*A. variegatum* BF, Gh, Ni
Covariate					
Weight (age proxy**)**	Prev	− **10.4 ± 4.8 10**^–3^*	33.8 ± 28.6 10^–4^	38.5 ± 25.6 10^–4^ ns	**31.6 ± 14.6 10**^–4^*
	Load	− **14.0 ± 5.2 10**^–3^**	25.7 ± 26.6 10^–4^	**38.6 ± 17.1 10**^**–4**^ *	**28.9 ± 14.6 10**^–4^*
Sex					
Female vs. male	Prev	− 0.35 ± 0.22 ns	0.46 ± 0.31 ns	− 0.17 ± 0.23 ns	− 0.26 ± 0.13 ns
	Load	− 0.63 ± 0.17***	0.28 ± 0.32 ns	0.03 ± 0.21 ns	− 0.22 ± 0.08**
Body condition	Prev	0.18 ± 0.13 ns	0.28 ± 0.15 ns	0.07 ± 0.14 ns	− 0.04 ± 0.09 ns
	Load	0.24 ± 0.08**	0.21 ± 0.17 ns	− 0.28 ± 0.11 *	0.03 ± 0.07 ns
Parasiticide		Gh, Ni		BF	Gh, BF
< 1 month vs. never	Prev	0.34 ± 0.19 ns		− 1.61 ± 0.71 *	− 0.26 ± 0.27 ns
	Load	0.13 ± 0.13 ns		− 2.82 ± 0.62 ***	− 0.04 ± 0.17 ns
1–2 months vs. never	Prev	0.07 ± 0.65 ns		NA	1.16 ± 0.60 ns
	Load	0.05 ± 0.53 ns		NA	0.09 ± 0.25 ns
> 2 months vs. never	Prev	NA		0.11 ± 0.82 ns	− 0.38 ± 0.81 ns
	Load	NA		− 0.87 ± 0.66 ns	− 1.57 ± 0.71*
Treated vs. never vs. never	Prev	0.32 ± 0.20 ns		− 0.81 ± 0.59 ns	− 0.26 ± 0.25 ns
	Load	0.11 ± 0.14 ns		− 1.54 ± 0.40 ***	− 0.11 ± 0.15 ns

Numbers in bold are linked to 'statistically significant' effects

Generalised estimation equations that model the tick species’ prevalence (levels: 0, 1) and infestation loads in cattle of smallholder rural areas with transhumance husbandry (Burkina Faso: 15.7%, Ghana: 54.5% and Nigeria: 24.2% of the total number of animals investigated). For a given taxon, only countries with a prevalence of at least 10% were included. Individual body weight and body condition were mean-centered at farm level (nested within country districts). Effects of parasiticides were tested only in Ghana and Burkina Faso (80.5% and 26.1% of the animals treated, respectively) only. Pre(valence): model estimates reflect the probability that tick has level ‘1’ (logit link). Lo(ad): model estimates reflect the associations between number of ticks and a unit increase in explanatory variable (log link)

*Bf* Burkina, *Gh* Ghana, *Be* Benin, *Ni* Nigeria, *Et* Ethiopia, *Ug* Uganda, *Ta* Tanzania. *NA* not applicable, since no data available

***P < 0.001

**P < 0.01

*P < 0.05

*ns P* > 0.05

In contrast to the positive associations found with body weight, several tick species showed a negative association with body condition (a proxy for production effect) in their loads at individual cattle level: *H. rufipes* (communal, log: − 0.13 ± 0.05 ticks/unit; *Z* = − 2.59; *P* = 0.009; transhumance, log: − 0.28 ± 0.11 ticks/unit; *Z* = − 1.81; *P* = 0.07); *A. gemma* (communal, log: − 0.72 ± 0.23 ticks/unit; *Z *= − 3.13; *P* = 0.0017). Again, for *R. microplus* in transhumance setting the opposite pattern with body condition score was observed (log: 0.24 ± 0.08; *Z* = 2.94; *P* = 0.003). Regarding tick prevalence, only *A. gemma* (communal, log: − 0.34 ± 0.09 ticks/unit; *Z* = − 3.70; *P* = 0.0002) showed a significant association with body condition.

Parasiticides had negative effects on tick loads (in communal settings, almost all effect sizes were negative), especially in *R. microplus* (log_treated-never_: − 0.74 ± 0.25; *Z *= 3.03; *P* = 0.0024) and *R. decoloratus* (log_treated-never_: − 0.98 ± 0.21; *Z* = 3.08; *P* = 0.0021). However, also in *A. variegatum* and *H. rufipes* (both communal and transhumance) less strong but statistically significant effects on loads as well as prevalence were observed (Table 4, 5) depending on the time since treatment. We emphasise that only a subset of countries could be used for the testing of parasiticide effects, given the unbalanced treatments (see Table 1).

### Co-occurrence of ticks at the individual level

Within the subpopulation of tick-infested cattle, proportions of co-infested individuals (i.e. different tick taxa feeding on the same host individual) significantly varied among countries (range: 49.22% to 98.56%; *χ*2 = 1525.31; df = 6; *P* < 0.001; Tables 2, 3) and were overall higher in East Africa (79.60%) compared to West Africa (64.73%). Highest levels of co-infestations were found in Uganda (98.56% of all infested cattle; Additional file 1: Table S4). Tanzania, another country with high tick diversity (Shannon’s Index: 1.82), also showed high levels of co-infestation (81.82%).

Likely because of the lower tick biodiversity in West African cattle, the number of tick combinations on the same host was much lower compared to East Africa (Additional file 1: Tables S4, S5). The combination *A. variegatum* × *R. microplus* was high in both Ghana (41.39%) and Benin (39.17%) and turned out to be the most common in Nigeria (6.92%) as well. *Amblyomma variegatum* × *H. rufipes* (5.68%) was the most common combination in Burkina Faso, where overall the level of co-infestations was (very) low (19.25%). In East Africa, the combination of ticks on the same host (overall) most often found was *A. variegatum* × *R. microplus* × *R. appendiculatus* × *R. evertsi evertsi* (15.62%), mainly because of the high proportion of this combination in Uganda (36.32%) where several of the lower orders within this combination were common as well (55.36%). *Amblyomma variegatum* × *R. decoloratus* (8.33%) was the most common combination in Tanzania and *A. variegatum* × *R. decoloratus* (9.57%) in Ethiopia.

## Discussion

The main objective of the study was to document the most important cattle ticks in seven sub-Saharan African countries and to identify potential extrinsic and intrinsic ecological risk factors for tick infestation. We guided the data collection via a rigorous pre-defined protocol and thus did an analysis on standardised data. Our study therefore embodies a relatively up-to-date status of cattle ticks in small-scale rural livestock production systems. Moreover, by respecting the hierarchical structure of the data in our analyses, we could investigate associations at the individual level—which is the level the least affected by ecological biases.

Around 70% of the animals sampled were infested with at least one tick species. Most of what follows will consider vector-competent ticks with economic importance. While several ticks occurred in high (e.g. *A. variegatum* vectoring *E. ruminantium*, *R. microplus* vectoring *B. bovis*) to medium (*H. rufipes* vectoring *A. marginale* and *R decoloratus* vectoring *B. bigemina* in addition) prevalences—of which most also showed significant spatial variation—other important vectors were nearly absent (e.g. *R. annulatus*, a tick considered to be vector of *A. marginale*, *B. bigemina* and *B. bovis*) or occurred focalised in one particular country or region, e.g. *R. pulchellus* in Ethiopia (another vector for *A. marginale*) and *R. appendiculatus* in Uganda and Tanzania (most important vector of *T. parva*). Surprisingly, the latter pathogenic agent causing East Coast fever was nearly absent based on molecular screenings of cattle blood [3], which is suggested to be due to high mortality in young and susceptible animals or the carrier status of recovered animals [6]. The most important finding was that *R. microplus* had spread to Uganda as described in the "Background" section, where half of the cattle sampled were infested with this tick species. This tick species is known for its invasive behaviour as seen in West Africa after its introduction from Brazil. Despite the recent introduction of *R. microplus* in Uganda, its high prevalence is not surprising, since the invasive character and establishment success of the species have been well observed in West Africa [14, 15, 30] and East Africa (see "Background" section). Despite the overt tick burden and reported control failure by local farmer communities in Africa, the economic impact of the recent introduction in West Africa and further expansion in Eastern and Southern Africa is not known. For most of the other tick species identified in the different countries, a status quo was observed. Because of the lack of standardised, detailed studies on tick species across the study area, it is difficult—not to say impossible—to verify displacement, density changes or local shifts in distribution.

Without having any thorough knowledge on local wildlife abundances and diversity, and micro-climatic conditions, several of the geographical patterns are more than likely the consequence of tick biology, habitat preferences as well as seasonality in host searching behaviour. It is generally assumed that most tick species show a seasonal abundance to synchronise the exposure of their most vulnerable life cycle stages (eggs and larvae) to the best climatic conditions of the year. Basically, adults of most tick species will start questing or actively start hunting for a host (in the case of *Amblyomma* spp. and *Hyalomma* spp.) at the start of the rainy season to produce eggs during the rains. At higher latitudes, seasonality is more pronounced because of the more distinct seasons: one or two well-defined rainy seasons interspersed by dry seasons. Although in our study seasonality has been incorporated in the analyses merely as a confounder, the temporal variation demonstrated contrasting country-specific tick profiles that are more than likely shaped by a range of biotic and abiotic conditions—most of which have not yet been determined. As a logical consequence, control measures will need to be locally tailor-made to optimise the use of financial resources of the resource-poor farmer communities to limit the development of resistance against acaricides, lower the morbidity and mortality and increase production.

Of the intrinsic exposure risk factors, the animal’s gender explained part of the variation in tick loads in that females had significantly lower tick loads than males. Besides behavioural differences between male and female in exposure risk (e.g. higher exploration and roaming in males), less grooming and/or immunosuppressive effects of testosterone may increase tick infestion levels (as well as tick-borne haemoparasites infection levels, see [3, 31, 32]). Body weight—a proxy for the cattle age but also skin surface—was positively associated with tick loads in six tick species. Higher cumulative exposure risk can be expected in bigger and older animals and/or higher infestation tolerance in the heavier animal. Alternatively, higher mortality in lighter animals may have led to the observed positive association with body weight, e.g. due to tick-borne pathogen mortality (*A. marginale* [8], *E. ruminantium* and *B. bigemina*) or the direct effects of tick infestations. In contrast, in individuals kept in transhumance settings, body weight was negatively associated with *R. microplus* loads and prevalence, which could be the effect of acquired immunity (more likely to develop with age) and carrier status of the animal, with higher levels of resistance in heavier animals. Without experiments, it is difficult to exclude alternative interpretations like age-related differences in exposure or innate resistance [32].

Lower tick loads of *H. rufipes* (both in communal and transhumance settings) and *A. gemma* were linked to animals with the highest body condition score (a proxy for production). Also here, both virulence/pathogenicity of (ecto-) parasites and/or higher anti-parasite resistance in hosts of higher body condition may have caused the associations. At the moment of infestation, animals may not necessarily be infected with the micro-parasites the ticks are transmitting. In most instances, there is a time lag between tick bite and systemic infections. This may be one of the reasons why in transhumance cattle we did not find an expected negative association between *R. microplus* and body condition score, despite in previous work [3]. *Babesia bovis* (vectored mainly by *R. microplus*) showed to be strongly negatively correlated with body condition scores translating in production losses. As this monotropic, one-host tick has developed resistant strains in several continents toward different classes of acaricides [33], it remains the most important acarological threat to cattle globally.

We observed measurable, but small, effects of parasiticides mainly on tick loads in members within each tick genus, despite the scattered information on types of products used, time since application and selection criterion. We advocate that highly standardised monitoring and (field) experimental studies would push forward the description of recommendations and application strategies of acaricide use in the rural areas under study. In addition, complementary work should be the documentation of the local levels of resistance against acaricides of most important tick species. For this, already first steps have been set for in vitro studies (larval packet test, unpublished data) and in vivo studies (on animals, control studies). At least 25 engorged females of *A. variegatum*, *R. appendiculatus* and *R. microplus* were collected at various sites and shipped to the Utrecht Centre for Tick-Borne Diseases (FAO Reference Centre). Each of the tick stocks was cycled for one generation and resulting larvae used to determine acaricide resistance status in the larval packet test. The most resistant stocks of each of the three species are investigated in resistance studies, both in vitro and in vivo.

One of the limitations of this observational study is the differentiation between correlation and causation in the above-mentioned associations between tick occurrence and the cattle’s general health measures. Assessing the pathology (e.g. anemia, icterus) would help to better understand whether the tick occurrences are linked to tick-borne pathogen harm and/or effects on physiology by the ticks themselves and thus would also result in a better assessment of local socio-economic impacts of the exposures tick-pathogen combinations. In the absence of experimental longitudinal data—controlling for ecological stressors affecting the animal’s health—cause and consequence in observations that include health impairments are impossible to disentangle.

The co-infestations found in individuals strongly suggest that cattle are susceptible and attractive to multiple tick species. The most striking outcome was the level of co-infestation in Uganda, where almost all individuals harboured multiple tick species. In this country, with high diversity of tick species, combinations of five species were commonly found. Further research is required to investigate whether tick combinations are higher (or lower) than expected by chance, i.e. whether biotic interactions would facilitate (or inhibit) co-feeding. This could be the result of variation in general susceptibility among individual animals but could also indicate transmission and/or proliferation facilitation or reduction. The pathways that lead to facilitation (e.g. gregarious feeding, increased exposure) and/or inhibition (e.g. competition) of co-feeding ticks at the level of cattle individuals can only be elucidated with experimental studies. Also its implications with respect to pathogen transmission cycles deserve further research, including the scenario of bridging tick vectors that transfer tick-borne pathogens from wildlife to cattle and humans. Moreover, the infestation of wild rodents by the tick *R. microplus* of domestic cattle has recently been demonstrated in Ivory Coast [34].

Regarding tick loads, a clear pattern was observed for almost all tick species: the majority of animals carry low tick numbers and a few animals high tick loads. Although this is not new or innovative science, hardly ever has this characteristic been used to optimise tick control. Theoretically, two options could be formulated: (i) control of ticks on a selected number of animals with above-average tick numbers (threshold control); (ii) culling of animals consistently carrying high tick loads (less adapted animals). The latter option might lead to genetically better adapted cattle, but production parameters should also be considered: the more resistant animals are often less productive based on their body weights. We argue that in smallholder farmer communities, those above-mentioned practices would not easily be adopted given the high economic value of each of the cattle, especially the larger (and thus heavier) individuals which turn out to have higher tick loads as well.

## Conclusions

This standardised surveillance underscores the importance of ticks of cattle in sub-Saharan Africa, with co-parasitism being the rule rather than the exception. Future studies could also include wildlife host surveys, tick densities in the off-host environment, detailed habitat characteristics (including vegetation), climatic conditions and specific resources that may support dense populations of ticks—and hence the circulation of tick-borne pathogens. Isolates of relevant tick strains should be evaluated for the effectiveness of different pharmaceutical and biological products, which may result in more effective control strategies. As transboundary movement of cattle between African countries is a major risk factor when governing vector-borne diseases in Africa, genetic population studies of relevant tick subpopulations may also provide further insights in the spread and invasion of tick populations and their pathogens. Also the genetics of the cattle (including cattle breeds) will further increase our knowledge about host susceptibility to both ticks and tick-borne pathogens. Integration of this knowledge with a good understanding of current complexities in socio-economic and climate changes will enable policymakers and scientists to provide prevention strategies.

### Supplementary Information

**Additional file 1: Table S1.** Spatio-temporal variation in tick prevalence in cattle of seven sub-Saharan countries. **Table S2.** Spatio-temporal variation in tick loads in cattle of seven sub-Saharan countries. **Table S3.** and **Table S4.** Distribution of (co-) infestations in cattle individuals.

**Additional file 2: **Protocol.

## Data Availability

The datasets generated and/or analysed during the current study are not publicly available due to Contract Research Organization agreements (data will be stored in the archives of Clinglobal, Mauritius), but are available from the corresponding author on reasonable request.
