# Correction: Tick communities of cattle in smallholder rural livestock production systems in sub-Saharan Africa

**DOI:** 10.1186/s13071-023-05895-x

**Published:** 2023-08-09

**Authors:** Dieter J. A. Heylen, Bersissa Kumsa, Elikira Kimbita, Mwiine Nobert Frank, Dennis Muhanguzi, Frans Jongejan, Safiou Bienvenu Adehan, Alassane Toure, Fred Aboagye-Antwi, Ndudim Isaac Ogo, Nick Julef, Josephus Fourie, Alec Evans, Joseph Byaruhanga, Maxime Madder

**Affiliations:** 1 Evolutionary Ecology Group, Department of Biology, University of Antwerp, Wilrijk, Belgium; 2 Interuniversity Institute for Biostatistics and Statistical Bioinformatics, Hasselt University, Diepenbeek, Belgium; 3 Department of Parasitology, College of Veterinary Medicine and Agriculture, Addis Ababa University, Bishoftu, Ethiopia; 4 Department of Veterinary Microbiology and Parasitology, College of Veterinary Medicine and Biomedical Sciences, Sokoine University of Agriculture, 3019 Morogoro, Tanzania; 5 Department of Bio-molecular Resources and Bio-Laboratory Sciences (BBS), College of Veterinary Medicine, Makerere University, Kampala, Uganda; 6 Department of Veterinary Tropical Diseases, Faculty of Veterinary Science, University of Pretoria, Onderstepoort, South Africa; 7 Zootechnical, Veterinary and Halieutic Research Laboratory (LRZVH), National Institute of Agricultural Research (INRAB), 01 BP 884 Cotonou, Benin; 8 Université Nangui Abrogoua, UFR Sciences de la Nature, 02 BP 801 Abidjan 02, Côte d’Ivoire; 9 Department of Animal Biology and Conservation Science, School of Biological Sciences, College of Basic and Applied Sciences, University of Ghana, Legon-Accra, Ghana; 10 National Veterinary Research Institute, Vom, Plateau State Nigeria; 11 Bill and Melinda Gates Foundation, Seattle, WA USA; 12 Clinvet International Pty (Ltd), 1479 Talmadge Hill South, Waverly, NY 14892 USA; 13 Clinglobal, B03/04, The Tamarin Commercial Hub, Jacaranda Avenue, Tamarin, 90903 Mauritius; 14 Department of Veterinary Pharmacy, Clinics and Comparative Medicine, School of Veterinary Medicine and Animal Resources, Research Center for Tropical Diseases and Vector Control (RTC), College of Veterinary Medicine, Animal Resources and Biosecurity, Makerere University, Kampala, Uganda

**Correction: Parasites & Vectors (2023) 16:206** 10.1186/s13071-023-05801-5

Following publication of the original article, the authors flagged that a species was incorrectly named in the Results subsection of the Abstract: instead of ‘*Rhipicephalus microplus* is known…', it said ‘*Rhipicephalus appendiculatus* is known…' The published article [1] has since been corrected. The authors thank you for reading this erratum and apologize for any inconvenience caused.

## References

[1] Heylen DJ, Kumsa B, Kimbita E, Frank MN, Muhanguzi D, Jongejan F, Adehan SB, Toure A, Aboagye-Antwi F, Ogo NI, Juleff N (2023). Tick communities of cattle in smallholder rural livestock production systems in sub-Saharan Africa. Parasit Vectors.

