# Publisher Correction: Tick communities of cattle in smallholder rural livestock production systems in sub-Saharan Africa

**DOI:** 10.1186/s13071-023-06034-2

**Published:** 2023-11-16

**Authors:** Dieter J. A. Heylen, Bersissa Kumsa, Elikira Kimbita, Mwiine Nobert Frank, Dennis Muhanguzi, Frans Jongejan, Safiou Bienvenu Adehan, Alassane Toure, Fred Aboagye‑Antwi, Ndudim Isaac Ogo, Nick Juleff, Josephus Fourie, Alec Evans, Joseph Byaruhanga, Maxime Madder

**Affiliations:** 1 Evolutionary Ecology Group, Department of Biology, University of Antwerp, Wilrijk, Belgium; 2 Interuniversity Institute for Biostatistics and Statistical Bioinformatics, Hasselt University, Diepenbeek, Belgium; 3 Department of Parasitology, College of Veterinary Medicine and Agriculture, Addis Ababa University, Bishoftu, Ethiopia; 4 Department of Veterinary Microbiology and Parasitology, College of Veterinary Medicine and Biomedical Sciences, Sokoine University of Agriculture, 3019 Morogoro, Tanzania; 5 Department of Bio‑molecular Resources and Bio‑Laboratory Sciences (BBS), College of Veterinary Medicine, Makerere University, Kampala, Uganda; 6 Department of Veterinary Tropical Diseases, Faculty of Veterinary Science, University of Pretoria, Onderstepoort, South Africa; 7 National Institute of Agricultural Research (INRAB), Zootechnical, Veterinary and Halieutic Research Laboratory (LRZVH), 01 BP 884, Cotonou, Benin; 8 Universite Nangui Abrogoua, UFR Sciences de la Nature, 02 Bp 801, Abidjan 02, Côte d’Ivoire; 9 Department of Animal Biology and Conservation Science, School of Biological Sciences, College of Basic and Applied Sciences, University of Ghana, Legon‑Accra, Ghana; 10 National Veterinary Research Institute, Vom, Plateau State Nigeria; 11 Bill & Melinda Gates Foundation, Seattle, WA USA; 12 Clinvet International Pty (Ltd), 1479 Talmadge Hill South, Waverly, NY 14892 USA; 13 Clinglobal, B03/04, The Tamarin Commercial Hub, Jacaranda Avenue, Tamarin, 90903 Mauritius; 14 Research Center for Tropical Diseases and Vector Control (RTC), Department of Veterinary Pharmacy, Clinics and Comparative Medicine, School of Veterinary Medicine and Animal Resources, College of Veterinary Medicine, Animal Resources and Biosecurity, Makerere University, Kampala, Uganda

**Publisher Correction: Parasites & Vectors (2023) 16(1):206** 10.1186/s13071-023-05801-5

Following publication of the original article [1], it was brought to the journal’s attention that a number of corrections provided by the authors during the proofing of their article had not been implemented. These corrections have now been implemented in the published article, and the details of the corrections can be seen in Additional file 1, linked to this erratum. The publisher thanks you for reading this erratum and apologizes for this processing error.

### Supplementary Information

**Additional file 1.** Corrections implemented post-publication.
