# Supplementary material for: Tick communities of cattle in smallholder rural livestock production systems in sub-Saharan Africa
Source: Parasit Vectors. 2023 Jun 19;16:206. doi: 10.1186/s13071-023-05801-5 (PMC10280850; doi:10.1186/s13071-023-05801-5)
Supplement: Supplementary file 1 — Additional file 1: Table S1. Spatio-temporal variation in tick prevalence in cattle of seven sub-Saharan countries. Table S2. Spatio-temporal variation in tick loads in cattle of seven sub-Saharan countries. Table S3. and Table S4. Distribution of (co-) infestations in cattle individuals. [file 13071_2023_5801_MOESM1_ESM.docx]

Additional File 1

Table S1. Spatio-temporal variation in tick prevalence in cattle of seven Sub-Saharan countries.

| Visit 1 | 2 | Burkina Faso (%) | | Ghana (%) | | Benin (%) | | Nigeria (%) | | Ethiopia (%) | | Uganda (%) | | Tanzania (%) | |
| --- | --- | --- | --- | --- | --- | --- | --- | --- | --- | --- | --- | --- | --- | --- | --- |
| 3 | 4 |  |  |  |  |  |  |  |  |  |  |  |  |  |  |
| ***A.gemma*** | | 0.00 | 0.00 | 0.00 | 0.00 | 0.00 | 0.00 | 0.00 | 0.00 | 0.00 | 12.92 | 0.00 | 0.00 | 0.42 | 0.41 |
|  | | 0.00 | 0.00 | 0.00 | 0.00 | 0.00 | 0.00 | 0.00 | 0.00 | 16.67 | 12.92 | 0.00 | 0.00 | 0.00 | 0.00 |
| **overall** | |  | **0.00** |  | **0.00** |  | **0.00** |  | **0.00** |  | **10.61** |  | **0.00** |  | **0.21** |
| ***A.variegatum*** | | 22.92 | 7.92 | 68.06 | 34.94 | 47.08 | 35.28 | 29.58 | 24.60 | 0.00 | 8.75 | 85.77 | 92.12 | 70.42 | 67.89 |
|  | | 16.32 | 25.13 | 71.15 | 48.29 | 85.77 | 43.84 | 14.46 | 30.13 | 32.92 | 38.33 | 94.61 | 93.33 | 15.00 | 5.39 |
| **overall** | |  | **17.54** |  | **55.96** |  | **50.88** |  | **24.66** |  | **19.98** |  | **91.38** |  | **39.81** |
| ***H.albiparmatum*** | | 0.00 | 0.00 | 0.38 | 0.00 | 0.00 | 0.00 | 0.00 | 0.00 | 0.00 | 0.00 | 0.00 | 0.00 | 3.33 | 1.22 |
|  | | 0.00 | 0.00 | 0.00 | 0.00 | 0.00 | 0.00 | 0.00 | 0.00 | 0.00 | 0.00 | 0.00 | 0.00 | 0.00 | 0.00 |
| **overall** | |  | **0.00** |  | **0.10** |  | **0.00** |  | **0.00** |  | **0.00** |  | **0.00** |  | **1.14** |
| ***H.impressum*** | | 2.08 | 0.00 | 0.00 | 0.00 | 0.00 | 0.00 | 0.00 | 0.00 | 0.00 | 0.00 | 0.00 | 0.00 | 0.00 | 0.00 |
|  | | 0.00 | 0.00 | 0.00 | 0.00 | 0.00 | 0.00 | 0.00 | 0.00 | 0.00 | 0.00 | 0.00 | 0.00 | 0.00 | 0.00 |
| **overall** | |  | **0.47** |  | **0.00** |  | **0.00** |  | **0.00** |  | **0.00** |  | **0.00** |  | **0.00** |
| ***H.rufipes*** | | 21.25 | 15.00 | 12.55 | 2.01 | 0.00 | 0.00 | 0.00 | 0.00 | 0.00 | 19.17 | 0.00 | 0.00 | 19.58 | 26.83 |
|  | | 34.47 | 14.36 | 11.07 | 0.85 | 0.00 | 0.00 | 0.00 | 0.00 | 7.08 | 3.75 | 0.00 | 0.00 | 32.92 | 9.54 |
| **overall** | |  | **23.32** |  | **6.81** |  | **0.00** |  | **0.00** |  | **7.49** |  | **0.00** |  | **22.23** |
| ***R.lunulatus*** | | 0.00 | 0.00 | 0.00 | 0.00 | 0.00 | 0.00 | 0.00 | 0.00 | 0.00 | 0.00 | 0.00 | 0.00 | 0.00 | 0.00 |
|  | | 0.00 | 0.00 | 0.00 | 0.00 | 0.00 | 0.00 | 0.00 | 0.00 | 0.00 | 11.25 | 0.00 | 0.00 | 0.00 | 0.00 |
| **overall** | |  | **0.00** |  | **0.00** |  | **0.00** |  | **0.00** |  | **2.81** |  | **0.00** |  | **0.00** |
| ***R.annulatus*** | | 0.00 | 0.00 | 0.00 | 0.00 | 1.67 | 1.67 | 0.00 | 0.00 | 0.00 | 0.00 | 0.00 | 0.00 | 0.00 | 0.00 |
|  | | 0.00 | 0.00 | 0.00 | 0.00 | 0.00 | 0.00 | 0.00 | 0.00 | 0.00 | 0.00 | 0.00 | 0.00 | 0.00 | 0.00 |
| **overall** | |  | **0.00** |  | **0.00** |  | **0.88** |  | **0.00** |  | **0.00** |  | **0.00** |  | **0.00** |
| ***R.appendiculatus*** | | 0.00 | 0.00 | 0.00 | 0.00 | 0.00 | 0.00 | 0.00 | 0.00 | 0.00 | 0.00 | 94.86 | 99.59 | 26.67 | 25.61 |
|  | | 0.00 | 0.00 | 0.00 | 0.00 | 0.00 | 0.00 | 0.00 | 0.00 | 0.00 | 0.00 | 98.76 | 99.17 | 56.25 | 26.56 |
| **overall** | |  | **0.00** |  | **0.00** |  | **0.00** |  | **0.00** |  | **0.00** |  | **98.05** |  | **33.71** |
| ***R.decoloratus*** | | 0.00 | 0.00 | 0.38 | 0.00 | 0.00 | 0.00 | 17.50 | 19.76 | 0.00 | 11.67 | 0.00 | 0.00 | 28.75 | 27.24 |
|  | | 0.00 | 0.00 | 0.00 | 0.00 | 0.00 | 0.00 | 4.96 | 3.35 | 12.08 | 20.00 | 0.41 | 2.50 | 36.67 | 27.39 |
| **overall** | |  | **0.00** |  | **0.10** |  | **0.00** |  | **11.46** |  | **10.93** |  | **0.72** |  | **29.99** |
| Note: Farms in seven African countries (one-two districts each) were visited four times within a time window of 12 months. | | | | | | | | | | | | | | | |

Table S1 continued. Spatio-temporal variation in tick prevalence in cattle of seven Sub-Saharan countries.

| Visit 1 | 2 | Burkina Faso (%) | | Ghana (%) | | Benin (%) | | Nigeria (%) | | Ethiopia (%) | | Uganda (%) | | Tanzania (%) | |
| --- | --- | --- | --- | --- | --- | --- | --- | --- | --- | --- | --- | --- | --- | --- | --- |
| 3 | 4 |  |  |  |  |  |  |  |  |  |  |  |  |  |  |
| ***R.evertsi evertsi*** | | 0.00 | 0.00 | 0.00 | 0.00 | 0.00 | 0.00 | 0.00 | 0.00 | 0.00 | 15.00 | 58.50 | 65.15 | 26.67 | 20.73 |
|  | | 0.00 | 0.00 | 0.00 | 0.00 | 0.00 | 0.00 | 0.00 | 0.00 | 11.67 | 9.58 | 74.69 | 78.33 | 15.42 | 15.77 |
| **overall** | |  | **0.00** |  | **0.00** |  | **0.00** |  | **0.00** |  | **9.05** |  | **69.03** |  | **19.65** |
| ***R.geigyi*** | | 0.00 | 0.00 | 0.00 | 0.00 | 0.42 | 0.00 | 0.00 | 0.00 | 0.00 | 0.00 | 0.00 | 0.00 | 0.00 | 0.00 |
|  | | 0.00 | 0.00 | 0.00 | 0.00 | 0.00 | 0.00 | 0.00 | 0.00 | 0.00 | 0.00 | 0.00 | 0.00 | 0.00 | 0.00 |
| **overall** | |  | **0.00** |  | **0.00** |  | **0.09** |  | **0.00** |  | **0.00** |  | **0.00** |  | **0.00** |
| ***R.microplus*** | | 40.83 | 32.50 | 73.38 | 30.52 | 38.33 | 31.11 | 17.50 | 43.55 | 0.00 | 0.00 | 45.45 | 52.28 | 52.50 | 49.59 |
|  | | 1.84 | 46.15 | 28.46 | 38.89 | 81.30 | 55.48 | 2.89 | 1.26 | 0.00 | 0.00 | 41.91 | 65.00 | 32.50 | 17.43 |
| **overall** | |  | **25.88** |  | **43.24** |  | **49.74** |  | **16.51** |  | **0.00** |  | **51.08** |  | **38.06** |
| ***R.praetextatus*** | | 0.00 | 0.00 | 0.00 | 0.00 | 0.00 | 0.00 | 0.00 | 0.00 | 0.00 | 14.17 | 0.00 | 0.00 | 0.00 | 0.00 |
|  | | 0.00 | 0.00 | 0.00 | 0.00 | 0.00 | 0.00 | 0.00 | 0.00 | 0.00 | 0.00 | 0.00 | 0.00 | 0.00 | 0.00 |
| **overall** | |  | **0.00** |  | **0.00** |  | **0.00** |  | **0.00** |  | **3.54** |  | **0.00** |  | **0.00** |
| ***R.pravus*** | | 0.00 | 0.00 | 0.00 | 0.00 | 0.00 | 0.00 | 0.00 | 0.00 | 0.00 | 0.42 | 0.00 | 0.00 | 0.00 | 0.00 |
|  | | 0.00 | 0.00 | 0.00 | 0.00 | 0.00 | 0.00 | 0.00 | 0.00 | 0.00 | 0.00 | 0.00 | 0.00 | 0.00 | 0.00 |
| **overall** | |  | **0.00** |  | **0.00** |  | **0.00** |  | **0.00** |  | **0.10** |  | **0.00** |  | **0.00** |
| ***R.pulchellus*** | | 0.00 | 0.00 | 0.00 | 0.00 | 0.00 | 0.00 | 0.00 | 0.00 | 0.00 | 38.33 | 0.00 | 0.00 | 0.00 | 0.00 |
|  | | 0.00 | 0.00 | 0.00 | 0.00 | 0.00 | 0.00 | 0.00 | 0.00 | 42.08 | 40.83 | 0.00 | 0.00 | 0.00 | 0.00 |
| overall | |  | **0.00** |  | **0.00** |  | **0.00** |  | **0.00** |  | **30.28** |  | **0.00** |  | **0.00** |
| ***R.sanguineus*** | | 0.00 | 0.00 | 0.00 | 0.00 | 0.00 | 0.00 | 0.00 | 0.00 | 0.00 | 0.00 | 0.00 | 0.00 | 0.00 | 0.00 |
|  | | 0.00 | 0.51 | 0.00 | 0.00 | 0.00 | 0.00 | 0.00 | 0.00 | 0.00 | 0.00 | 0.00 | 0.00 | 0.00 | 0.00 |
| **overall** | |  | **0.09** |  | **0.00** |  | **0.00** |  | **0.00** |  | **0.00** |  | **0.00** |  | **0.00** |
| ***H.truncatum*** | | 10.83 | 6.25 | 0.00 | 0.00 | 0.00 | 0.00 | 21.67 | 6.85 | 0.00 | 24.17 | 0.00 | 0.00 | 0.00 | 0.00 |
|  | | 0.53 | 9.74 | 0.00 | 0.00 | 0.00 | 0.00 | 6.61 | 10.88 | 5.00 | 11.25 | 0.00 | 0.00 | 1.25 | 0.83 |
| **overall** | |  | **5.88** |  | **0.00** |  | **0.00** |  | **11.46** |  | **10.09** |  | **0.00** |  | **0.52** |
| Note: Farms in seven African countries (one-two districts each) were visited four times within a time window of 12 months. | | | | | | | | | | | | | | | |

Table S2. Spatio-temporal variation in tick loads in cattle of seven Sub-Saharan countries.

| Visit 1 | 2 | Burkina Faso (%) | | Ghana (%) | | Benin (%) | | Nigeria (%) | | Ethiopia (%) | | Uganda (%) | | Tanzania (%) | |
| --- | --- | --- | --- | --- | --- | --- | --- | --- | --- | --- | --- | --- | --- | --- | --- |
| 3 | 4 |  |  |  |  |  |  |  |  |  |  |  |  |  |  |
| ***A.gemma*** | | 0+0 | 0+0 | 0+0 | 0+0 | 0+0 | 0+0 | 0+0 | 0+0 | 0+0 | 1.23+3.95 | 0+0 | 0+0 | 0.03+0.41 | 0.02+0.38 |
|  | | 0+0 | 0+0 | 0+0 | 0+0 | 0+0 | 0+0 | 0+0 | 0+0 | 3.45+15.32 | 2.01+7.15 | 0+0 | 0+0 | 0+0 | 0+0 |
| **overall** | |  | **0+0** |  | **0+0** |  | **0+0** |  | **0+0** |  | **1.67+8.76** |  | **0+0** |  | **0.01+0.28** |
| ***A.variegatum*** | | 0.4+0.88 | 0.1+0.39 | 18.19+22.19 | 12.5+31.91 | 25.54+44.25 | 19.55+45.42 | 2.07+4.26 | 2.12+5.53 | 0+0 | 0.7+2.57 | 11.26+12.79 | 20.68+23.36 | 10.99+23.5 | 7.82+9.83 |
|  | | 0.7+1.87 | 1.34+2.86 | 21.91+31.31 | 7.06+10.19 | 43.84+48.84 | 40.03+68.81 | 0.68+1.92 | 2.96+6.48 | 15.37+33.38 | 19.47+37.89 | 42.49+47.42 | 36.11+34.94 | 1.14+3.39 | 0.59+3.18 |
| **overall** | |  | **0.62+1.77** |  | **15.11+26.18** |  | **31.32+53.79** |  | **1.95+4.92** |  | **8.88+26.67** |  | **27.42+34.42** |  | **5.15+13.64** |
| ***H.albiparmatum*** | | 0+0 | 0+0 | 0.07+1.17 | 0+0 | 0+0 | 0+0 | 0+0 | 0+0 | 0+0 | 0+0 | 0+0 | 0+0 | 0.16+1.01 | 0.04+0.36 |
|  | | 0+0 | 0+0 | 0+0 | 0+0 | 0+0 | 0+0 | 0+0 | 0+0 | 0+0 | 0+0 | 0+0 | 0+0 | 0+0 | 0+0 |
| **overall** | |  | **0+0** |  | **0.02+0.6** |  | **0+0** |  | **0+0** |  | **0+0** |  | **0+0** |  | **0.05+0.54** |
| ***H.impressum*** | | 0.03+0.23 | 0+0 | 0+0 | 0+0 | 0+0 | 0+0 | 0+0 | 0+0 | 0+0 | 0+0 | 0+0 | 0+0 | 0+0 | 0+0 |
|  | | 0+0 | 0+0 | 0+0 | 0+0 | 0+0 | 0+0 | 0+0 | 0+0 | 0+0 | 0+0 | 0+0 | 0+0 | 0+0 | 0+0 |
| **overall** | |  | **0.01+0.11** |  | **0+0** |  | **0+0** |  | **0+0** |  | **0+0** |  | **0+0** |  | **0+0** |
| ***H.rufipes*** | | 0.47+1.13 | 0.26+0.74 | 1.4+4.47 | 0.19+1.54 | 0+0 | 0+0 | 0+0 | 0+0 | 0+0 | 2.11+6.87 | 0+0 | 0+0 | 0.99+2.64 | 3.54+10.2 |
|  | | 2.56+4.23 | 0.94+2.69 | 1.23+4.47 | 0.04+0.42 | 0+0 | 0+0 | 0+0 | 0+0 | 0.64+3.51 | 0.19+1.08 | 0+0 | 0+0 | 3.91+8.58 | 0.64+2.22 |
| **overall** | |  | **1.26+3.03** |  | **0.74+3.36** |  | **0+0** |  | **0+0** |  | **0.73+3.98** |  | **0+0** |  | **2.28+7.05** |
| ***R.lunulatus*** | | 0+0 | 0+0 | 0+0 | 0+0 | 0+0 | 0+0 | 0+0 | 0+0 | 0+0 | 0+0 | 0+0 | 0+0 | 0+0 | 0+0 |
|  | | 0+0 | 0+0 | 0+0 | 0+0 | 0+0 | 0+0 | 0+0 | 0+0 | 0+0 | 1.25+5.69 | 0+0 | 0+0 | 0+0 | 0+0 |
| **overall** | |  | **0+0** |  | **0+0** |  | **0+0** |  | **0+0** |  | **0.31+2.89** |  | **0+0** |  | **0+0** |
| ***R.annulatus*** | | 0+0 | 0+0 | 0+0 | 0+0 | 1.33+13.74 | 0.34+3.76 | 0+0 | 0+0 | 0+0 | 0+0 | 0+0 | 0+0 | 0+0 | 0+0 |
|  | | 0+0 | 0+0 | 0+0 | 0+0 | 0+0 | 0+0 | 0+0 | 0+0 | 0+0 | 0+0 | 0+0 | 0+0 | 0+0 | 0+0 |
| **overall** | |  | **0+0** |  | **0+0** |  | **0.39+6.66** |  | **0+0** |  | **0+0** |  | **0+0** |  | **0+0** |
| ***R.appendiculatus*** | | 0+0 | 0+0 | 0+0 | 0+0 | 0+0 | 0+0 | 0+0 | 0+0 | 0+0 | 0+0 | 125.22+70.9 | 150.73+71.01 | 1.89+4.53 | 2.5+6.96 |
|  | | 0+0 | 0+0 | 0+0 | 0+0 | 0+0 | 0+0 | 0+0 | 0+0 | 0+0 | 0+0 | 181.16+95.83 | 196.01+110.45 | 6.1+9.07 | 3.27+7.69 |
| **overall** | |  | **0+0** |  | **0+0** |  | **0+0** |  | **0+0** |  | **0+0** |  | **162.78+92.5** |  | **3.43+7.42** |
| ***R.decoloratus*** | | 0+0 | 0+0 | 0.09+1.38 | 0+0 | 0+0 | 0+0 | 0.87+2.12 | 6.02+20.4 | 0+0 | 2.61+16.81 | 0+0 | 0+0 | 3.14+10.29 | 2.67+5.88 |
|  | | 0+0 | 0+0 | 0+0 | 0+0 | 0+0 | 0+0 | 0.24+1.36 | 0.12+0.68 | 0.7+2.31 | 3.09+10.6 | 0.01+0.21 | 0.09+0.63 | 8+19.5 | 3.08+6.78 |
| **overall** | |  | **0+0** |  | **0.02+0.71** |  | **0+0** |  | **1.85+10.6** |  | **1.6+10.06** |  | **0.03+0.33** |  | **4.21+12.05** |
| Note: Farms in seven African countries (one-two districts each) were visited four times within a time window of 12 months. | | | | | | | | | | | | | | | |

Table S2 continued. Spatio-temporal variation in tick loads in cattle of seven Sub-Saharan countries.

| Visit 1 | 2 | Burkina Faso (%) | | Ghana (%) | | Benin (%) | | Nigeria (%) | | Ethiopia (%) | | Uganda (%) | | Tanzania (%) | |
| --- | --- | --- | --- | --- | --- | --- | --- | --- | --- | --- | --- | --- | --- | --- | --- |
| 3 | 4 |  |  |  |  |  |  |  |  |  |  |  |  |  |  |
| ***R.evertsi evertsi*** | | 0+0 | 0+0 | 0+0 | 0+0 | 0+0 | 0+0 | 0+0 | 0+0 | 0+0 | 1.07+3.43 | 3.31+4.63 | 10.04+12.5 | 2.36+5.92 | 1.09+2.6 |
|  | | 0+0 | 0+0 | 0+0 | 0+0 | 0+0 | 0+0 | 0+0 | 0+0 | 0.79+2.69 | 0.51+1.93 | 11.39+13.5 | 13.33+18.79 | 0.99+2.8 | 1.25+4.29 |
| **overall** | |  | **0+0** |  | **0+0** |  | **0+0** |  | **0+0** |  | **0.59+2.41** |  | **9.44+13.79** |  | **1.42+4.15** |
| ***R.geigyi*** | | 0+0 | 0+0 | 0+0 | 0+0 | 0.04+0.57 | 0+0 | 0+0 | 0+0 | 0+0 | 0+0 | 0+0 | 0+0 | 0+0 | 0+0 |
|  | | 0+0 | 0+0 | 0+0 | 0+0 | 0+0 | 0+0 | 0+0 | 0+0 | 0+0 | 0+0 | 0+0 | 0+0 | 0+0 | 0+0 |
| **overall** | |  | **0+0** |  | **0+0** |  | **0.01+0.26** |  | **0+0** |  | **0+0** |  | **0+0** |  | **0+0** |
| ***R.microplus*** | | 2.5+4.76 | 1.12+2.2 | 16.6+23.21 | 4.14+13.23 | 46.31+85.34 | 50.4+149.37 | 1.63+5.24 | 8.5+19.33 | 0+0 | 0+0 | 5.54+12.4 | 5.25+9.29 | 43.51+78.21 | 38.32+65.31 |
|  | | 0.07+0.61 | 3.77+5.29 | 2.57+5.01 | 4.33+8.17 | 92+73.25 | 49.38+71.43 | 0.09+0.5 | 0.03+0.28 | 0+0 | 0+0 | 5.54+11.79 | 15.56+30.84 | 5.4+11.23 | 1.95+6.19 |
| **overall** | |  | **1.55+3.67** |  | **7.07+15.49** |  | **58.27+106.56** |  | **2.61+10.71** |  | **0+0** |  | **7.94+18.65** |  | **22.37+54.66** |
| ***R.praetextatus*** | | 0+0 | 0+0 | 0+0 | 0+0 | 0+0 | 0+0 | 0+0 | 0+0 | 0+0 | 1.47+5.3 | 0+0 | 0+0 | 0+0 | 0+0 |
|  | | 0+0 | 0+0 | 0+0 | 0+0 | 0+0 | 0+0 | 0+0 | 0+0 | 0+0 | 0+0 | 0+0 | 0+0 | 0+0 | 0+0 |
| **overall** | |  | **0+0** |  | **0+0** |  | **0+0** |  | **0+0** |  | **0.37+2.72** |  | **0+0** |  | **0+0** |
| ***R.pravus*** | | 0+0 | 0+0 | 0+0 | 0+0 | 0+0 | 0+0 | 0+0 | 0+0 | 0+0 | 0.01+0.16 | 0+0 | 0+0 | 0+0 | 0+0 |
|  | | 0+0 | 0+0 | 0+0 | 0+0 | 0+0 | 0+0 | 0+0 | 0+0 | 0+0 | 0+0 | 0+0 | 0+0 | 0+0 | 0+0 |
| **overall** | |  | **0+0** |  | **0+0** |  | **0+0** |  | **0+0** |  | **0+0.08** |  | **0+0** |  | **0+0** |
| ***R.pulchellus*** | | 0+0 | 0+0 | 0+0 | 0+0 | 0+0 | 0+0 | 0+0 | 0+0 | 0+0 | 4.29+7.33 | 0+0 | 0+0 | 0+0 | 0+0 |
|  | | 0+0 | 0+0 | 0+0 | 0+0 | 0+0 | 0+0 | 0+0 | 0+0 | 25.55+43.61 | 12.73+21.26 | 0+0 | 0+0 | 0+0 | 0+0 |
| **overall** | |  | **0+0** |  | **0+0** |  | **0+0** |  | **0+0** |  | **10.63+26.35** |  | **0+0** |  | **0+0** |
| ***R.sanguineus*** | | 0+0 | 0+0 | 0+0 | 0+0 | 0+0 | 0+0 | 0+0 | 0+0 | 0+0 | 0+0 | 0+0 | 0+0 | 0+0 | 0+0 |
|  | | 0+0 | 0.02+0.21 | 0+0 | 0+0 | 0+0 | 0+0 | 0+0 | 0+0 | 0+0 | 0+0 | 0+0 | 0+0 | 0+0 | 0+0 |
| **overall** | |  | **0+0.09** |  | **0+0** |  | **0+0** |  | **0+0** |  | **0+0** |  | **0+0** |  | **0+0** |
| ***H.truncatum*** | | 0.17+0.6 | 0.11+0.5 | 0+0 | 0+0 | 0+0 | 0+0 | 1.56+3.53 | 0.43+1.97 | 0+0 | 3.06+6.97 | 0+0 | 0+0 | 0+0 | 0+0 |
|  | | 0.02+0.3 | 0.58+2.02 | 0+0 | 0+0 | 0+0 | 0+0 | 0.43+2.06 | 0.67+2.24 | 0.38+1.96 | 0.8+2.8 | 0+0 | 0+0 | 0.14+1.37 | 0.04+0.61 |
| **overall** | |  | **0.18+0.98** |  | **0+0** |  | **0+0** |  | **0.77+2.57** |  | **1.06+4.05** |  | **0+0** |  | **0.04+0.75** |
| Note: Farms in seven African countries (one-two districts each) were visited four times within a time window of 12 months. | | | | | | | | | | | | | | | |

Table S3. Distribution of (co-)infestations in cattle individuals of West Africa.

| **Co- infestations West Africa** | **Overall** | **Burkina Faso** | **Ghana** | **Benin** | **Nigeria** |
| --- | --- | --- | --- | --- | --- |
| *A.variegatum x R.microplus* | 25.87 | 4.42 | 41.81 | 39.17 | 6.92 |
| *R.microplus* | 23.50 | 34.23 | 15.64 | 23.33 | 21.17 |
| *A.variegatum* | 21.24 | 15.46 | 32.16 | 14.29 | 26.62 |
| *H.rufipes* | 6.86 | 27.44 | 1.61 | 0.00 | 0.00 |
| *R.Boophilus* spp. | 3.85 | 0.32 | 0.00 | 11.38 | 0.00 |
| *A.variegatum x R.Boophilus* spp. | 3.60 | 0.00 | 0.15 | 10.71 | 0.00 |
| *R.decoloratus* | 2.71 | 0.00 | 0.00 | 0.00 | 15.30 |
| *H.truncatum* | 2.52 | 3.15 | 0.00 | 0.00 | 10.06 |
| *A.variegatum x H.rufipes* | 2.08 | 5.68 | 2.92 | 0.00 | 0.00 |
| *A.variegatum x H.truncatum* | 1.41 | 1.10 | 0.00 | 0.00 | 6.50 |
| *A.variegatum x R.microplus x H.rufipes* | 1.30 | 0.63 | 4.53 | 0.00 | 0.00 |
| *A.variegatum x R.decoloratus* | 1.19 | 0.00 | 0.00 | 0.00 | 6.71 |
| *R.decoloratus x H.truncatum* | 0.82 | 1.42 | 0.00 | 0.00 | 2.73 |
| *A.variegatum x R.microplus x H.truncatum* | 0.67 | 0.79 | 0.00 | 0.00 | 2.73 |
| *H.rufipes x H.truncatum* | 0.56 | 2.37 | 0.00 | 0.00 | 0.00 |
| *R.microplus x H.rufipes* | 0.48 | 1.10 | 0.88 | 0.00 | 0.00 |
| *R.microplus x R.annulatus* | 0.15 | 0.00 | 0.00 | 0.45 | 0.00 |
| *A.variegatum x H.rufipes x H.truncatum* | 0.15 | 0.63 | 0.00 | 0.00 | 0.00 |
| *H.rufipes x H.impressum* | 0.11 | 0.47 | 0.00 | 0.00 | 0.00 |
| *R.decoloratus x H.truncatum* | 0.11 | 0.00 | 0.00 | 0.00 | 0.63 |
| *A.variegatum x R.annulatus* | 0.11 | 0.00 | 0.00 | 0.33 | 0.00 |
| *A.variegatum x R.decoloratus x H.truncatum* | 0.11 | 0.00 | 0.00 | 0.00 | 0.63 |
| *H.impressum* | 0.04 | 0.16 | 0.00 | 0.00 | 0.00 |
| *R.annulatus* | 0.04 | 0.00 | 0.00 | 0.11 | 0.00 |
| *R.microplus x H.albiparmatum* | 0.04 | 0.00 | 0.15 | 0.00 | 0.00 |
| *R.microplus x H.rufipes x H.truncatum* | 0.04 | 0.16 | 0.00 | 0.00 | 0.00 |
| *R.microplus x R.annulatus x R.geigyi* | 0.04 | 0.00 | 0.00 | 0.11 | 0.00 |
| *A.variegatum x H.rufipes x H.impressum* | 0.04 | 0.16 | 0.00 | 0.00 | 0.00 |
| *A.variegatum x R.microplus x H.rufipes x H.truncatum* | 0.04 | 0.16 | 0.00 | 0.00 | 0.00 |
| *A.variegatum x R.microplus x R.sanguineus* | 0.04 | 0.16 | 0.00 | 0.00 | 0.00 |
| *A.variegatum x R.microplus x R.annulatus* | 0.04 | 0.00 | 0.00 | 0.11 | 0.00 |
| *A.variegatum x R.microplus x R.decoloratus* | 0.04 | 0.00 | 0.15 | 0.00 | 0.00 |
| **N° Infested (%)** | **2698(64.73)** | **634(60.09)** | **691(68.68)** | **896(78.73)** | **477(49.22)** |
| **Co-infestation** | **39.04** | **19.25** | **50.59** | **50.88** | **26.85** |
| Note: Prevalences refer to the number of host individuals with one or more tick species infestations. From top to bottom: most to least frequently observed (combinations of) infestations. (Percentages sum to 100% for each). | | | | | |

Table S4. Distribution of (co-)infestations in cattle individuals of East Africa.

| **Co- infestations East Africa (1)** | **Overall** | **Ethiopia** | **Uganda** | **Tanzania** |
| --- | --- | --- | --- | --- |
| *A.variegatum x R.microplus x R.appendiculatus x R.evertsi.evertsi* | 15.62 | 0.00 | 36.32 | 1.53 |
| *A.variegatum x R.appendiculatus x R.evertsi.evertsi* | 12.12 | 0.00 | 28.51 | 0.76 |
| *A.variegatum x R.microplus x R.appendiculatus* | 6.97 | 0.00 | 12.80 | 4.83 |
| *A.variegatum x R.appendiculatus* | 6.06 | 0.00 | 14.05 | 0.64 |
| *R.decoloratus* | 5.84 | 3.19 | 0.00 | 14.89 |
| *R.pulchellus* | 5.28 | 21.63 | 0.00 | 0.00 |
| *A.variegatum* | 4.72 | 13.65 | 0.10 | 3.94 |
| *A.variegatum x R.decoloratus* | 4.46 | 8.33 | 0.00 | 7.12 |
| *R.appendiculatus* | 2.38 | 0.00 | 2.19 | 4.33 |
| *A.gemma x R.pulchellus* | 2.34 | 9.57 | 0.00 | 0.00 |
| *A.variegatum x R.microplus* | 1.99 | 0.00 | 0.10 | 5.73 |
| *R.microplus x R.appendiculatus* | 1.69 | 0.00 | 0.52 | 4.33 |
| *R.appendiculatus x R.evertsi.evertsi* | 1.56 | 0.00 | 2.91 | 1.02 |
| *R.microplus* | 1.51 | 0.00 | 0.00 | 4.45 |
| *A.variegatum x R.microplus x R.appendiculatus x H.rufipes* | 1.51 | 0.00 | 0.00 | 4.45 |
| *A.variegatum x R.decoloratus x R.evertsi.evertsi* | 1.34 | 1.06 | 0.00 | 3.18 |
| *R.microplus x R.appendiculatus x R.evertsi.evertsi* | 1.17 | 0.00 | 1.46 | 1.65 |
| *R.decoloratus x R.appendiculatus* | 1.04 | 0.00 | 0.00 | 3.05 |
| *A.variegatum x R.microplus x H.rufipes* | 1.00 | 0.00 | 0.00 | 2.93 |
| *R.microplus x R.appendiculatus x H.rufipes* | 1.00 | 0.00 | 0.00 | 2.93 |
| *R.evertsi.evertsi* | 0.95 | 1.77 | 0.00 | 1.53 |
| *R.evertsi.evertsi x R.pulchellus* | 0.91 | 3.72 | 0.00 | 0.00 |
| *A.variegatum x R.microplus x R.appendiculatus x R.evertsi.evertsi x H.rufipes* | 0.91 | 0.00 | 0.00 | 2.67 |
| *R.pulchellus x H.rufipes* | 0.87 | 3.55 | 0.00 | 0.00 |
| *A.variegatum x R.microplus x R.evertsi.evertsi* | 0.78 | 0.00 | 0.10 | 2.16 |
| *A.variegatum x R.evertsi.evertsi* | 0.69 | 1.06 | 0.21 | 1.02 |
| *R.microplus x H.rufipes* | 0.56 | 0.00 | 0.00 | 1.65 |
| *H.rufipes* | 0.52 | 1.06 | 0.00 | 0.76 |
| *R.appendiculatus x H.rufipes* | 0.52 | 0.00 | 0.00 | 1.53 |
| *R.microplus x R.appendiculatus x R.evertsi.evertsi x H.rufipes* | 0.52 | 0.00 | 0.00 | 1.53 |
| *H.truncatum* | 0.48 | 1.95 | 0.00 | 0.00 |
| *R.pulchellus x H.truncatum* | 0.48 | 1.95 | 0.00 | 0.00 |
| *R.decoloratus x H.rufipes* | 0.43 | 0.00 | 0.00 | 1.27 |
| *A.gemma x R.evertsi.evertsi x R.pulchellus* | 0.43 | 1.77 | 0.00 | 0.00 |
| *A.variegatum x R.decoloratus x H.rufipes* | 0.39 | 0.53 | 0.00 | 0.76 |
| *A.variegatum x R.microplus x R.evertsi.evertsi x H.rufipes* | 0.39 | 0.00 | 0.00 | 1.15 |

Table S4 continued. Distribution of (co-)infestations in cattle individuals of East Africa.

| **Co- infestations East Africa (2)** | **Overall** | **Ethiopia** | **Uganda** | **Tanzania** |
| --- | --- | --- | --- | --- |
| *A.variegatum x R.microplus x R.decoloratus* | 0.35 | 0.00 | 0.00 | 1.02 |
| *R.appendiculatus x R.evertsi.evertsi x H.rufipes* | 0.35 | 0.00 | 0.00 | 1.02 |
| *R.decoloratus x R.appendiculatus x H.rufipes* | 0.35 | 0.00 | 0.00 | 1.02 |
| *A.variegatum x R.decoloratus x R.lunulatus* | 0.35 | 1.42 | 0.00 | 0.00 |
| *A.variegatum x R.decoloratus x R.appendiculatus* | 0.35 | 0.00 | 0.10 | 0.89 |
| *A.gemma x R.pulchellus x H.truncatum* | 0.35 | 1.42 | 0.00 | 0.00 |
| *A.gemma x R.pulchellus x H.rufipes* | 0.30 | 1.24 | 0.00 | 0.00 |
| *R.praetextatus x H.truncatum* | 0.26 | 1.06 | 0.00 | 0.00 |
| *R.decoloratus x R.evertsi.evertsi* | 0.26 | 0.00 | 0.00 | 0.76 |
| *A.variegatum x R.lunulatus* | 0.26 | 1.06 | 0.00 | 0.00 |
| *A.gemma* | 0.26 | 1.06 | 0.00 | 0.00 |
| *A.variegatum x H.truncatum* | 0.22 | 0.89 | 0.00 | 0.00 |
| *A.variegatum x R.decoloratus x H.truncatum* | 0.22 | 0.89 | 0.00 | 0.00 |
| *R.lunulatus* | 0.22 | 0.89 | 0.00 | 0.00 |
| *R.evertsi.evertsi x R.pulchellus x H.truncatum* | 0.22 | 0.89 | 0.00 | 0.00 |
| *R.evertsi.evertsi x R.pulchellus x H.rufipes* | 0.22 | 0.89 | 0.00 | 0.00 |
| *A.variegatum x R.appendiculatus x H.rufipes* | 0.22 | 0.00 | 0.00 | 0.64 |
| *H.rufipes x H.truncatum* | 0.17 | 0.71 | 0.00 | 0.00 |
| *R.praetextatus* | 0.17 | 0.71 | 0.00 | 0.00 |
| *R.praetextatus x H.rufipes* | 0.17 | 0.71 | 0.00 | 0.00 |
| *R.evertsi.evertsi x H.rufipes* | 0.17 | 0.18 | 0.00 | 0.38 |
| *R.decoloratus x R.praetextatus* | 0.17 | 0.71 | 0.00 | 0.00 |
| *R.microplus x R.evertsi.evertsi* | 0.17 | 0.00 | 0.00 | 0.51 |
| *A.variegatum x R.decoloratus x R.evertsi.evertsi x H.rufipes* | 0.17 | 0.00 | 0.00 | 0.51 |
| *A.gemma x R.evertsi.evertsi x R.pulchellus x H.truncatum* | 0.17 | 0.71 | 0.00 | 0.00 |
| *A.variegatum x H.rufipes* | 0.13 | 0.18 | 0.00 | 0.25 |
| *R.decoloratus x R.evertsi.evertsi x H.truncatum* | 0.13 | 0.35 | 0.00 | 0.13 |
| *R.microplus x R.decoloratus* | 0.13 | 0.00 | 0.00 | 0.38 |
| *A.variegatum x R.lunulatus x H.truncatum* | 0.13 | 0.53 | 0.00 | 0.00 |
| *A.variegatum x R.evertsi.evertsi x H.truncatum* | 0.13 | 0.53 | 0.00 | 0.00 |
| *A.variegatum x R.decoloratus x R.appendiculatus x R.evertsi.evertsi* | 0.13 | 0.00 | 0.10 | 0.25 |
| *A.variegatum x R.microplus x R.decoloratus x R.evertsi.evertsi* | 0.13 | 0.00 | 0.00 | 0.38 |
| *A.gemma x R.pulchellus x H.rufipes x H.truncatum* | 0.13 | 0.53 | 0.00 | 0.00 |
| *A.gemma x A.variegatum x R.pulchellus* | 0.13 | 0.53 | 0.00 | 0.00 |
| *R.decoloratus x H.truncatum* | 0.09 | 0.35 | 0.00 | 0.00 |
| *R.pulchellus x H.rufipes x H.truncatum* | 0.09 | 0.35 | 0.00 | 0.00 |

Table S4 continued. Distribution of (co-)infestations in cattle individuals of East Africa.

| **Co- infestations East Africa (3)** | **Overall** | **Ethiopia** | **Uganda** | **Tanzania** |
| --- | --- | --- | --- | --- |
| *R.decoloratus x H.rufipes x H.truncatum* | 0.09 | 0.35 | 0.00 | 0.00 |
| *R.decoloratus x R.praetextatus x H.truncatum* | 0.09 | 0.35 | 0.00 | 0.00 |
| *R.decoloratus x R.appendiculatus x R.evertsi.evertsi* | 0.09 | 0.00 | 0.00 | 0.25 |
| *R.microplus x R.appendiculatus x R.evertsi.evertsi x H.truncatum* | 0.09 | 0.00 | 0.00 | 0.25 |
| *R.microplus x R.decoloratus x R.appendiculatus* | 0.09 | 0.00 | 0.00 | 0.25 |
| *R.microplus x R.decoloratus x R.appendiculatus x R.evertsi.evertsi* | 0.09 | 0.00 | 0.21 | 0.00 |
| *A.variegatum x R.praetextatus x H.rufipes x H.truncatum* | 0.09 | 0.35 | 0.00 | 0.00 |
| *A.variegatum x R.evertsi.evertsi x H.rufipes* | 0.09 | 0.00 | 0.00 | 0.25 |
| *A.variegatum x R.evertsi.evertsi x R.lunulatus* | 0.09 | 0.35 | 0.00 | 0.00 |
| *A.variegatum x R.decoloratus x R.lunulatus x H.truncatum* | 0.09 | 0.35 | 0.00 | 0.00 |
| *A.variegatum x R.decoloratus x R.evertsi.evertsi x H.rufipes x H.albiparmatum* | 0.09 | 0.00 | 0.00 | 0.25 |
| *A.variegatum x R.microplus x R.appendiculatus x R.evertsi.evertsi x H.albiparmatum* | 0.09 | 0.00 | 0.00 | 0.25 |
| *A.variegatum x R.microplus x R.appendiculatus x R.evertsi.evertsi x H.rufipes x H.albiparmatum* | 0.09 | 0.00 | 0.00 | 0.25 |
| *A.variegatum x R.microplus x R.decoloratus x R.appendiculatus* | 0.09 | 0.00 | 0.10 | 0.13 |
| *A.variegatum x R.microplus x R.decoloratus x R.appendiculatus x R.evertsi.evertsi* | 0.09 | 0.00 | 0.21 | 0.00 |
| *A.gemma x R.evertsi.evertsi x R.pulchellus x H.rufipes* | 0.09 | 0.35 | 0.00 | 0.00 |
| *A.gemma x A.variegatum x R.microplus* | 0.09 | 0.00 | 0.00 | 0.25 |
| *A.variegatum x R.Boophilus* spp. | 0.04 | 0.00 | 0.00 | 0.13 |
| *A.variegatum x H.rufipes x H.truncatum* | 0.04 | 0.18 | 0.00 | 0.00 |
| *R.praetextatus x H.rufipes x H.truncatum* | 0.04 | 0.18 | 0.00 | 0.00 |
| *R.praetextatus x R.pulchellus* | 0.04 | 0.18 | 0.00 | 0.00 |
| *R.praetextatus x R.pulchellus x H.truncatum* | 0.04 | 0.18 | 0.00 | 0.00 |
| *R.pravus x R.pulchellus* | 0.04 | 0.18 | 0.00 | 0.00 |
| *R.evertsi.evertsi x R.pulchellus x H.rufipes x H.truncatum* | 0.04 | 0.18 | 0.00 | 0.00 |
| *R.evertsi.evertsi x R.praetextatus x H.rufipes* | 0.04 | 0.18 | 0.00 | 0.00 |
| *R.evertsi.evertsi x R.praetextatus x R.pulchellus x H.truncatum* | 0.04 | 0.18 | 0.00 | 0.00 |
| *R.appendiculatus x H.albiparmatum* | 0.04 | 0.00 | 0.00 | 0.13 |
| *R.appendiculatus x H.truncatum* | 0.04 | 0.00 | 0.00 | 0.13 |
| *R.Boophilus* spp*. x H.rufipes* | 0.04 | 0.00 | 0.00 | 0.13 |
| *R.decoloratus x R.praetextatus x H.rufipes x H.truncatum* | 0.04 | 0.18 | 0.00 | 0.00 |
| *R.microplus x R.evertsi.evertsi x H.rufipes* | 0.04 | 0.00 | 0.00 | 0.13 |
| *R.microplus x R.appendiculatus x H.rufipes x H.albiparmatum* | 0.04 | 0.00 | 0.00 | 0.13 |
| *R.microplus x R.appendiculatus x R.evertsi.evertsi x H.rufipes x H.truncatum* | 0.04 | 0.00 | 0.00 | 0.13 |
| *R.microplus x R.Boophilus* spp. | 0.04 | 0.00 | 0.00 | 0.13 |
| *R.microplus x R.decoloratus x R.evertsi.evertsi x H.rufipes* | 0.04 | 0.00 | 0.00 | 0.13 |
| *R.microplus x R.decoloratus x R.appendiculatus x H.rufipes* | 0.04 | 0.00 | 0.00 | 0.13 |

Table S4 continued. Distribution of (co-)infestations in cattle individuals of East Africa.

| **Co-infestations East Africa (4)** | **Overall** | **Ethiopia** | **Uganda** | **Tanzania** |
| --- | --- | --- | --- | --- |
| *A.variegatum x R.praetextatus* | 0.04 | 0.18 | 0.00 | 0.00 |
| *A.variegatum x R.praetextatus x H.truncatum* | 0.04 | 0.18 | 0.00 | 0.00 |
| *A.variegatum x R.praetextatus x R.pulchellus x H.rufipes x H.truncatum* | 0.04 | 0.18 | 0.00 | 0.00 |
| *A.variegatum x R.evertsi.evertsi x R.pulchellus* | 0.04 | 0.18 | 0.00 | 0.00 |
| *A.variegatum x R.evertsi.evertsi x R.pulchellus x H.rufipes x H.truncatum* | 0.04 | 0.18 | 0.00 | 0.00 |
| *A.variegatum x R.evertsi.evertsi x R.praetextatus x R.pulchellus x H.rufipes x H.truncatum* | 0.04 | 0.18 | 0.00 | 0.00 |
| *A.variegatum x R.evertsi.evertsi x R.lunulatus x H.truncatum* | 0.04 | 0.18 | 0.00 | 0.00 |
| *A.variegatum x R.appendiculatus x H.albiparmatum* | 0.04 | 0.00 | 0.00 | 0.13 |
| *A.variegatum x R.Boophilus* spp. *x R.appendiculatus x R.evertsi.evertsi* | 0.04 | 0.00 | 0.00 | 0.13 |
| *A.variegatum x R.decoloratus x R.pulchellus* | 0.04 | 0.18 | 0.00 | 0.00 |
| *A.variegatum x R.decoloratus x R.praetextatus x H.truncatum* | 0.04 | 0.18 | 0.00 | 0.00 |
| *A.variegatum x R.decoloratus x R.evertsi.evertsi x H.truncatum* | 0.04 | 0.18 | 0.00 | 0.00 |
| *A.variegatum x R.microplus x H.rufipes x H.albiparmatum* | 0.04 | 0.00 | 0.00 | 0.13 |
| *A.variegatum x R.microplus x R.appendiculatus x H.rufipes x H.albiparmatum* | 0.04 | 0.00 | 0.00 | 0.13 |
| *A.variegatum x R.microplus x R.decoloratus x H.rufipes* | 0.04 | 0.00 | 0.00 | 0.13 |
| *A.gemma x R.praetextatus x R.pulchellus* | 0.04 | 0.18 | 0.00 | 0.00 |
| *A.gemma x R.evertsi.evertsi* | 0.04 | 0.18 | 0.00 | 0.00 |
| *A.gemma x R.evertsi.evertsi x R.pulchellus x H.rufipes x H.truncatum* | 0.04 | 0.18 | 0.00 | 0.00 |
| *A.gemma x A.variegatum x R.pulchellus x H.truncatum* | 0.04 | 0.18 | 0.00 | 0.00 |
| *A.gemma x A.variegatum x R.pulchellus x H.rufipes* | 0.04 | 0.18 | 0.00 | 0.00 |
| **Co-infested cows (%)** | **2311(79.60)** | **564(58.68)** | **961(98.56)** | **786(81.28)** |
| **Infested individuals** | **77.66** | **54.09** | **97.7** | **70.11** |
| Note: Prevalences refer to the number of host individuals with one or more tick species infestations. From top to bottom: most to least frequently observed (combinations of) infestations. (Percentages sum to 100% for each). | | | | |
